# Ameliorative impacts of gamma-aminobutyric acid (GABA) on seedling growth, physiological biomarkers, and gene expression in eight wheat (*Triticum aestivum* L.) cultivars under salt stress

**DOI:** 10.1186/s12870-024-05264-5

**Published:** 2024-06-26

**Authors:** Abdelfattah Badr, Mostafa M. Basuoni, Mohamed Ibrahim, Yossry E. Salama, Sawsan Abd-Ellatif, Elsayed S. Abdel Razek, Khaled E. Amer, Amira A. Ibrahim, Ehab M. Zayed

**Affiliations:** 1 Botany and Microbiology Department, Faculty of Science, Helwan University, Cairo, Egypt; 2 Botany and Microbiology Department, Faculty of Science (Boys), Al-Azhar University, Cairo, 11884 Egypt; 3 Department of Botany, Faculty of Science, Ain Shams University, Cairo, Egypt; 4 Crop Science Department, Faculty of Agriculture, Damanhour University, Beheira Governorate, Damanhour, 22516 Egypt; 5 Bioprocess Development Department, Genetic Engineering and Biotechnology Research Institute (GEBRI), City of the Scientific Research and Technological Application (SRTA-City), New Borg El-Arab, Alexandria 21934 Egypt; 6 Livestock Research Department, City of Scientific Research and Technological Applications (SRTA-City), Arid Lands Cultivation Research Institute (ALCRI), New Borg El-Arab, Alexandria 21934 Egypt; 7 Botany and Microbiology Department, Faculty of Science, Arish University, Al-Arish, 45511 Egypt; 8 Cell Study Research Department, Field Crops Research Institute, Agricultural Research Center (ARC), Giza, 12619 Egypt

**Keywords:** GABA, Wheat, Photosynthesis, Salt stress biomarkers, Antioxidant enzymes, Differential gene expression

## Abstract

Plants spontaneously accumulate γ-aminobutyric acid (GABA), a nonprotein amino acid, in response to various stressors. Nevertheless, there is limited knowledge regarding the precise molecular mechanisms that plants employ to cope with salt stress. The objective of this study was to investigate the impact of GABA on the salt tolerance of eight distinct varieties of bread wheat (*Triticum aestivum* L.) by examining plant growth rates and physiological and molecular response characteristics. The application of salt stress had a detrimental impact on plant growth markers. Nevertheless, the impact was mitigated by the administration of GABA in comparison to the control treatment. When the cultivars Gemmiza 7, Gemmiza 9, and Gemmiza 12 were exposed to GABA at two distinct salt concentrations, there was a substantial increase in both the leaf chlorophyll content and photosynthetic rate. Both the control wheat cultivars and the plants exposed to salt treatment and GABA treatment showed alterations in stress-related biomarkers and antioxidants. This finding demonstrated that GABA plays a pivotal role in mitigating the impact of salt treatments on wheat cultivars. Among the eight examined kinds of wheat, CV. Gemmiza 7 and CV. Gemmiza 11 exhibited the most significant alterations in the expression of their TaSOS1 genes. CV. Misr 2, CV. Sakha 94, and CV. Sakha 95 exhibited the highest degree of variability in the expression of the NHX1, DHN3, and GR genes, respectively. The application of GABA to wheat plants enhances their ability to cope with salt stress by reducing the presence of reactive oxygen species (ROS) and other stress indicators, regulating stomatal aperture, enhancing photosynthesis, activating antioxidant enzymes, and upregulating genes involved in salt stress tolerance.

**Supplementary Information:**

The online version contains supplementary material available at 10.1186/s12870-024-05264-5.

## Highlights

1. Gamma-aminobutyric acid (GABA) has been proven to promote seedling growth in wheat varieties exposed to salt stress. As a result, there was enhanced root growth, longer shoot length, and improved overall health of the seedlings.

2. GABA has been shown to enhance physiological indicators (chlorophyll levels and enhanced relative water content) in wheat varieties under salt stress conditions and minimize membrane impairment, all of which indicate improved stress resistance and general plant well-being.

3. GABA therapy has been found to increase gene expression in wheat cultivars subjected to salt stress. This includes the upregulation of stress-responsive genes and the downregulation of genes associated with negative stress responses, ultimately leading to improved resilience and adaptation to harsh growing conditions.

4. The impact of GABA on seedling growth, physiological biomarkers, and gene expression can change depending on the unique wheat cultivar. Each wheat variety may show distinct reactions to GABA therapy, emphasizing the need for cultivar-specific studies and customized strategies to optimize the advantages of GABA in reducing salt stress in wheat farming.

**Supplementary Information:**

The online version contains supplementary material available at 10.1186/s12870-024-05264-5.

## Introduction

Climate change has affected agroecosystem productivity through many mechanisms, such as elevated mean temperatures, increased occurrence and intensity of droughts, and greater soil salinity [1]. Soil salinity, an abiotic stress, has caused a decline in agricultural productivity in various parts of the world. It is predicted to worsen [2, 3]. Salt stress is the most challenging abiotic stress for plants because it severely impacts several cellular functions, such as germination, anatomy, physiology, biochemistry, and yield [4]. A decrease in agricultural productivity directly results in food insecurity, causing greater unpredictability in food production and reduced expected yields in multiple countries [5]. The global availability of cultivable land has decreased by at least 20% due to the combined effects of salt stress caused by climate change and human activities [6].

Bread wheat exhibits a certain degree of salt tolerance, but excessive salinity can cause a substantial decrease in yield, exceeding 50% [7]. Hence, the cultivation of wheat in saline areas is limited by the use of tactics to alleviate the detrimental impacts of salinity stress on its growth and yield. By using agronomic practices such as the application of mineral gypsum, organic amendments, and effective drainage systems, it is feasible to mitigate adverse effects. The user’s response is not fully provided. Approximately 85% of the irrigated land in Iran is affected by many limitations, including salinity [7]. Salinity has impacted approximately 14% of irrigated land in Pakistan, leading to substantial decreases in crop productivity. Salinity accounts for approximately 64% of the overall crop output reductions in the country [8]. Severe surface salinity impacts more than 2.5 million hectares of irrigated land in different parts of Pakistan. Excessive soil salinity can greatly hinder the process of seed germination and the growth of plants. This is mostly caused by variables such as excessive osmotic potential and the toxic effects of certain ions. Salinity exerts a substantial influence on various aspects of cellular function, including cell components, photosynthetic mechanisms, membrane structure, reactive oxygen species formation, and enzymatic activity. Ultimately, these consequences impede the development and productivity of crops. Research indicates that the process of seed germination and the initial growth of plants are more vulnerable to the negative effects of salt stress [9]. Salinity affects approximately 6% of the Earth’s land, with 20% of arable land and 33% of irrigated area being particularly vulnerable [10].

According to Hazman et al. [1], if sea levels rise, the soil salinity of agricultural land in northern Egypt might increase, and the severity of floods on Mediterranean beaches could deteriorate. Agroecosystem variables are highly susceptible to widely recognized climate change and anthropogenic salinity issues. Approximately 30% of Egypt’s cultivable land is affected by salinity, primarily due to the extensive use of inorganic fertilizers over several decades [11]. Salt can impede plant development, performance, and yield through several mechanisms, such as physiological drought resulting from osmosis, nutritional shortages, and specific ion toxicity, notably sodium ions [12–15]. Excessive sodium ions in salt soil or excessively salinized irrigation water can decrease water absorption efficiency, increase water leakage, and reduce root cell osmotic potential [16]. According to Arif et al. [17], sodium ions can disturb vital cellular functions in plants by creating an imbalance in the absorption of potassium and other crucial ions by target cells. Under high salt conditions, Na ions induce ionic toxicity and osmotic stress [15, 16].

The presence of salt stress promotes the production of dangerous amounts of reactive oxygen species (ROS), such as superoxide, hydroxyl, and hydrogen peroxide. This phenomenon occurs as a result of oxidative stress [1, 9, 18]. Plants disturb their carbon metabolism and produce reactive oxygen species (ROS) when they are exposed to salt stress [19]. Hussain et al. [20] discovered that plants experience a decrease in the exchange of CO_2_ gas when they are subjected to elevated salt concentrations. This results in a reduction in the abundance of oxidized NADP^+^ and a redirection of electron movement toward molecular oxygen (O_2_). The final stage of this process occurs when O2^• −^, sometimes referred to as superoxide anion, is produced. The activation of NADPH oxidases on the cell membrane results in the accumulation of hydrogen peroxide in the apoplast. The Fenton-/Haber-Weiss reaction can convert hydrogen peroxide into highly reactive hydroxyl radicals (^•^OH) [1, 21]. A recent study on plants revealed that reactive oxygen species (ROS) play dual roles by facilitating specific vital biological cellular pathways [22, 23]. Plants generate malondialdehyde (MDA) and other byproducts of lipid peroxidation due to oxidative stress produced by the excessive production and accumulation of reactive oxygen species (ROS). These chemicals are essential for plants to withstand stress [24]. Apel and Hirt [25], Fehér et al. [26], and Mansoor et al. [27] reported that an overabundance of reactive oxygen species (ROS) can lead to harm to nucleic acids, protein oxidation, enzyme inhibition, and the onset of programmed cell death, ultimately leading to cell death. Salinity stress triggers osmotic stress and ion toxicity by facilitating the absorption of Na^+^ ions and decreasing the Na^+^/K^+^ ratio as a result of diminished osmotic potential in the roots of plants. Furthermore, these disparities in ions have a significant influence on the assimilation and mobility of essential ions in certain cells, hence impeding basic processes and functions in plants [28]. Elevated salt levels impede the growth of plants, restrict plant growth, impede reproductive development, and ultimately diminish agricultural yield [29]. Salinity alters the structural components of cells, interferes with the machinery involved in photosynthesis, damages membrane structure, increases the production of reactive oxygen species, and reduces enzymatic activity. These effects ultimately hinder the growth and productivity of crops [30].

Furthermore, studies have demonstrated that subjecting seeds to salt stress during the germination phase is essential for assessing a wide range of species [31]. Seedling studies have demonstrated that wheat crops progress through three main stages of establishment: germination, emergence, and early seedling growth. These time intervals are particularly susceptible to the impacts of salt [32]. Salinity stress has a major negative impact on plant development and productivity, resulting in a large loss in agricultural output [33].

Photosynthesis is the main physiological process that is essential for the life of plants, and it is mostly affected by external conditions. Salt stress affects the growth of plants by impacting various characteristics, including shoot length, root length, root fresh weight, and shoot fresh weight [34]. Roots, which are located in the soil and are responsible for the absorption of water, play a critical role in assessing the impact of salt stress. Therefore, both the lengths of the roots and the length of the shoots are important factors in this evaluation. Elevated salinity adversely affects the rate of seed germination, resulting in a reduction in the density of plants [35].

Wheat (*Triticum aestivum* L.) is the top food crop globally due to its domestication and high nutritional value for humans [36, 37]. Aprile et al. [38] predicted that the demand for wheat would increase by 60% by the year 2050. According to Dhanda et al. [39], the harmful effects of repeated drought stress are thought to be most significant during growth and development in agricultural ecosystems in arid and semiarid climates. Climate change can lead to a combination of living and nonliving stress conditions, further decreasing wheat productivity. This crop is already impacted by high temperatures, a lack of water, and high salt levels [40, 41]. Asseng et al. [42] reported that various climate models suggested that stressful conditions could result in a 6% reduction in wheat output. However, a recent study by Shew et al. [43] on 72 distinct wheat cultivars revealed that the average wheat yield decreased by 8.5% under a constant warming scenario of + 1 °C. The percentage increases to 18.4% and 28.5% for situations with temperatures of + 2 °C and + 3 °C, respectively. A recent study suggested that exchanging genetic material among wheat breeding programs can help reduce the impacts of climate change [43].

Furthermore, wheat employs several reactions and mechanisms, such as biochemical, physiological, morphological, and molecular mechanisms, to respond to salinity stress and facilitate the acquisition and maintenance of crucial cellular processes and pathways [4, 23]. Liao et al. [44] discovered that stomatal conductance is the immediate reaction to salinity stress tolerance. On the other hand, Munns and Tester [16] and Duarte-Delgado et al. [45] observed that plants with long-term salt tolerance exhibit a harmonious accumulation of ions and water. Genetics, different forms of salinity, and the amount of exposure all contribute to the variable levels of salt tolerance observed in different wheat genotypes. The presence of excessive levels of salt in the environment has an osmotic effect, which negatively affects the ability of seeds to germinate and roots to emerge. This is problematic because it prevents plants from obtaining nutrients for healthy growth [46]. Furthermore, wheat grown under salinity stress exhibited restricted crop output due to ion toxicity, osmotic stress, and mineral deficiencies, as observed by Trono and Pecchioni [47]. To improve breeding programs and reduce the decrease in crop output caused by high salt levels, the genetic diversity among different wheat varieties was examined to identify cultivars that are tolerant to salt stress [47, 48].

GABA has been extensively researched for four decades due to its involvement in the adaptive response to abiotic stress. It is an effective compatible osmolyte and has a significant role in plant development [49]. The role of GABA in connection with stress has been indicated by indirect evidence. Genetic and physiological research conducted by Kinnersley and Lin [50] suggested that plants may possess receptors similar to GABA receptors. Ma et al. [49] and Shelp et al. [51] investigated the function of receptors in connection to the role of GABA in signaling and the significance of appropriate osmolytes in plant stress responses, respectively.

Considering the previously indicated benefits of GABA, we focused specifically on the impact of salt stress on wheat cultivars and how GABA mitigates this stress. This study aimed to investigate whether the alleviating effects of GABA and its ability to improve salt tolerance may be achieved in eight different wheat cultivars by altering their morphological, metabolic, and molecular responses. We ensured the correlation among all the integrated data.

## Materials and methods

### Plant materials

The eight bread wheat cultivars used in this study were provided by the Field Crops Research Institute, Agricultural Research Center (ARC), Giza, Egypt. The cultivars included Sakha 94, Sakha 95, Gemmiza 7, Gemmiza 9, Gemmiza 10, Gemmiza 11, Gemmiza 12, and Misr2. A full identification file was kindly provided along with the cultivar information (Table 1). The wheat grains were identified and assessed for their pedigree by Dr. Ehab M. Zayed, an experienced researcher specializing in field crop biotechnology. The voucher specimens were kept at the official grain stores of the Field Crops Research Institute, Agricultural Research Center (ARC), Giza Governorate, Egypt. High-quality gamma-aminobutyric acid (GABA) with a purity of at least 0.99 was obtained from Sigma‒Aldrich (Germany).

**Table 1:** Name, pedigree, selection history and year of release of the bread wheat cultivars and used in the current study

Ser #	Name	Pedigree	Selection History	Year of release
**1**	SAKHA 94	OPATA / RAYON // KAUZ	CMBW90Y3180-0TOPM-3Y-010 M-010 M-010Y-10 M-015Y-0Y-0AP-0 S-0EGY	2004
**2**	SAKHA 95	PASTOR // SITE / MO /3/ CHEN / AEGILOPS SQUARROSA (TAUS) // BCN /4/ WBLL1.	CMA01Y00158S-040POY-040 M-030ZTM-040SY-26 M-0Y-0SY-0 S-0EGY	-
**3**	GEMMIZA 7	CMH 74 A.630 / 5X // SERI 82 /3/ AGENT	GM 4611-2GM-3GM-1GM-0GM-0EGY	2000
**4**	GEMMIZA 9	ALD “S” / HUAC // CMH 74 A. 630 / 5X	GM 4583-5GM-1GM-0GM-0EGY	2000
**5**	GEMMIZA 10	MAYA 74 “S”/ON//1160 − 147 /3/ BB / GLL /4 / CHAT"S” /5/ CROW “S”	CGM5820-3GM-1GM-2GM-0GM-0EGY	2004
**6**	GEMMIZA 11	BOW"S”/KVZ"S” // 7 C / SER182/3/GIZA168/SAKHA61	GM7892-2GM-1GM-2GM-1GM-0GM-0EGY	2011
**7**	GEMMIZA 12	OTUS /3/ SARA / THB // VEE	CMSS97Y00227S-5Y-010 M-010Y-010 M-2Y-1 M-0Y-0GM-0EGY	2013
**8**	MISR 2	SKAUZ / BAV92	CMSS96M03611S-1 M-010SY-010 M-010SY-8 M-0Y-0EGY	2014

### Pot experiment setup

The experiments were conducted in the greenhouse facility of SRTA city, Alexandria Governorate, Egypt, starting in March 2023. Wheat grains of all cultivars were sterilized, and 20 homogenous viable, healthy seeds were sown in each plastic pot (10 cm diameter × 12 cm depth). The pots were composed of clay soil and compost (4:1), supplemented with N, K, and P nutrients, and moistened with 1/4 strength Hoagland nutrient solution every two days for one week.

### Salt stress regimens and GABA application

One week after sowing, the plants that had germinated were thinned, and the number of germinated plants decreased from 20 to eight per pot. These plants were then grown for two weeks and irrigated with 50% Hoagland solution every three days. After three weeks of growth, the plants were separated into six groups. Group (C) consisted of control plants that were regularly irrigated with a 50% Hoagland solution every three days. The seedlings in the other five groups were subjected to five different treatments involving salinity stress and GABA application. Group S1 was irrigated with a 137 mM (8000 ppm) NaCl solution every three days for one week, which resulted in relatively low salinity stress. The S2 group experienced significant salinity stress after regular irrigation with a high-NaCl solution for one week. A group of plants were treated with a solution containing GABA at a concentration of 3 mg/L. The solution was prepared in 50% Hoagland solution and applied to the plants’ leaves every three days for a period of two weeks. Two groups, labeled GABA + S1 and GABA + S2, were created by combining the salinity treatments with GABA application. The experiment was conducted with four replicates for each treatment. The plants were carefully selected and cultivated in a controlled environment. The plants were placed in a greenhouse under specific conditions, including a photoperiod of 13/11 h (light/dark), an air relative humidity of 65 ± 5%, and a temperature of 25/18 ± 4 °C (day/night) during the pot experiments. Samples treated with GABA at 7 and 14 days of age were collected for future analysis.

### Seedling growth measurements

Samples were collected from four separate pots belonging to each treatment group after 7 and 14 days of GABA treatment to estimate growth characteristics. Three separate biological replicates were chosen and rinsed with dH_2_O to eliminate any soil or particles that may have struck them. The plant length (Ph) was measured immediately after harvest. The SFW and RFW were measured. In addition, the SDW and RDW were measured following a 48-hour oven-drying period at 80 °C. The final harvesting stage involved measuring the number of leaves per plant and the area.

### Estimation of chlorophyll and gas exchange parameters

The chlorophyll content of both treated and untreated plants was measured using spectrophotometry following the method described by Arnon [52]. The leaf samples, weighing 0.2 g, were placed in a 10 ml solution of acetone with 80% water content. Afterwards, the samples were centrifuged at 12,000 × g for 10 min using a Hermle Centrifuge made in Germany. A UV/VIS spectrophotometer from Genway, Japan, was used to measure the absorbance at wavelengths of 663 nm and 645 nm. To evaluate the net photosynthetic rate (Pn), stomatal conductance (Gs), and transpiration rate (Tr), we utilized infrared gas analyzer equipment (TPS-2, portable photosynthesis device, USA) to examine leaf samples from both treated and untreated plants.

### Quantifying leaf relative water content and leaf water potential

The calculation of the relative water content (RWC) was based on the research conducted by Arndta et al. [53] and Barr and Weatherley [54]. We used the following formula to calculate the RWC: The RWC percentage can be calculated using the formula [(FW - DW)/(TW - DW)] *100. In addition, the researchers used a pressure chamber to measure the leaf water potential in the fully expanded leaves of both the control and stressed plants following the method described by Scholander et al. [55]. The pressure chamber used was from PMS Instruments Company (USA). Leaves were collected from the second set of fully grown leaves on each plant.

### Estimation of stress-induced biomarkers

#### Hydrogen peroxide (H_2_O_2_)

We followed the method outlined by Najafi Kakavand et al. [56] to determine the H_2_O_2_ concentration. To summarize, trichloroacetic acid (TCA) was used to extract leaf samples, which were then centrifuged at 12,000 × g for 15 min. Next, half a milliliter of the enzyme wastewater was combined with half a milliliter of phosphate buffer (pH 7.0) containing potassium iodide (1 mM). With H_2_O_2_ as the standard, the absorbance of the reaction mixture was measured at 390 nm.

#### Malondialdehyde (MDA) measurement

Measuring the MDA concentration is a common method for indicating lipid peroxidation [57]. Heath and Packer [58] devised a technique for measuring the concentration of MDA in freshly harvested leaves. Their method involves a condensation reaction between two molecules of thiobarbitoric acid (Siga Aldrich, Germany) and one molecule of MDA. The rate of this reaction is influenced by factors such as temperature, pH, and TBA concentration. A deduction was made for the unspecified value at A600. To determine the MDA concentration, the difference between A532 and A600 nm was divided by the MDA molar extinction coefficient, which is 155 mM^− 1^ cm-1. The values obtained are reported as µmol g^− 1^ FW.

#### Measurement of electrolyte leakage

Following Sullivan [59], electrolyte leakage (EL) was quantified. To summarize, 20 new leaf discs were immersed in 10 ml of deionized water in a boiling tube, and their electrical conductivity (EC) was recorded and represented as EC1. Then, the electrical conductivity (EC2) was measured again after the tubes were heated at 55 °C for 30 min. After that, EC3 was measured after boiling the tissue homogenate for 10 min at 100 °C. The formula for calculating the EC was {EC2- EC1)/EC3*100, which represents the percentage of electrolyte leakage.

### Extraction and estimation of proline and total protein content

A study conducted by Bates et al. [60] involved the purification of proline from 500 mg of dry powdered tissue using 3% sulfosalicylic acid. Two milliliters of the supernatant was subjected to a reaction with 2 ml of glacial acetic acid and 2 ml of ninhydrin reagent in a water bath set at 100 °C for one hour. Afterward, extraction centrifugation was performed at 3000 × g for 20 min. The tubes were placed in a chilled bath, and the proline was extracted using toluene. In their study, Bates et al. [60] measured the optical density at 520 nm. The amount of soluble protein was determined using the Bradford [61] method with Folin phenol reagent. The absorbance was measured at 700 nm using bovine serum albumin (BSA) as the reference standard.

### Extraction and measurements of antioxidant enzymes

To extract antioxidant enzymes, a prechilled mortar and pestle were used to homogenize 1 g of fresh leaf tissue in 50 mM phosphate buffer (pH 7.0), which also included 1 mM EDTA and 1% polyvinylpyrrolidone. The mixture was centrifuged at 15,000 × g for 20 min at 4 °C. The extraction of enzymes was performed using the second supernatant. An assay mixture of 1.5 mL was prepared, consisting of sodium phosphate buffer (50 mM, pH 7.5), EDTA, L-methionine, NBT (75 µM), riboflavin, and the enzyme extract. This mixture was utilized to determine the activity of superoxide dismutase (SOD, EC 1.15.1.1). Absorbance measurements were taken at 560 nm to record the photochemical reductions of NBT. The light was switched off after 15 min of incubation, and the activity was measured as EU mg^− 1^ protein. Based on Luck’s research [62], the catalase test (CAT, EC1.11.1.6) was performed, with absorbance monitoring at 240 nm for a duration of 2 min. The extinction coefficient used in the calculation was 39.4 mM^− 1^ cm-^1^. A reaction mixture was prepared with 1 mL of potassium phosphate buffer (pH 7.0), 0.5 mM ascorbic acid, hydrogen peroxide, and enzyme extract to evaluate ascorbate peroxidase activity (APX, EC 1.11.1.11) [63]. A change in the absorbance at 290 nm was observed for 3 min. Nakano and Asada [64] reported that the extinction coefficient was 2.8 mM^− 1^ cm^− 1^.

### Gene expression analysis

Using the manufacturer’s procedure, a total of half a gram of each wheat cultivar was utilized to isolate the total mRNA. This was performed by employing the Plant RNA Kit from Sigma‒Aldrich. Following purification, the RNA was examined on a 1% agarose gel, and its quantity was measured using spectrophotometry. Each sample contained the following component in the reaction mixture: 10 µg of total RNA, with 5 µg being reverse transcribed. The mixture included 10 ml/µl oligo dT primer, 2.5 µl 5X buffer, 2.5 µl MgCl_2_, 2.5 µl 2.5 mM dNTPs, 4 µl oligo dT, 0.2 µl 5 units/µl reverse transcriptase from Promega, Germany, and 2.5 µl RNA. The thermal cycler PCR was programmed for RT‒PCR amplification at 42 °C for 1 h and 72 °C for 20 min. The Rotor-Gene 6000 system, a cutting-edge technology from Germany, was utilized to perform real-time PCR analysis. The analysis involved using 1 µL of diluted cDNA in triplicate, ensuring accurate and reliable results. The primers used for qRT‒PCR can be found in Table 2. For the analysis of gene expression, a SYBR® Green-based approach was used, utilizing primers for four different genes. These genes included one related to salt tolerance, one related to phytochelatin, one related to the Zn transporter, and one related to the housekeeping gene β-actin. The overall volume of the reaction was 20 µL. A small volume of template, a specific amount of SYBR Green Master Mix, reverse and forward primers, and sterile dist water were carefully added to the mixture. The reaction mixture was composed of water. The following PCR conditions were used: the temperature was increased to 95 °C for 15 min and then decreased to 60 °C for 30 s; this cycle was repeated 40 times. The CT values of the target gene were subtracted from the CT values of the β-actin gene to determine the ΔCT values. According to the research conducted by Livak and Schmittgen [65], the 2^−ΔΔCt^ method was utilized to calculate the relative gene expression.

**Table 2:** Oligonucleotide primers’ sequences used in qRT-PCR analysis

Gene Name		Sequence
**NHX1**	**F**	5´- CTCAAGGGTGACTACCAAGCA- 3´
	**R**	5´- CCAATGCATCCATCCCGAC- 3´
**DHN3**	**F**	5´- CATGGCGTCTACTGCTTGTA-3’
	**R**	5´- CAGAGGACTTGAACCCAGATAC-3’
**GR**	**F**	5´- GATGGAGGCTACTTGCTTTG- 3´
	**R**	5´- GCTAAGACCCACGACAGATA − 3´
**TaSOS1**	**F**	5´- GTTGTCGGTGAGGTCGGAGGG- 3´
	**R**	5´- CATCTTCTCCTACCGCCCTGC- 3´
**β-Actin**	**F**	5´-GTGCCCATTTACGAAGGATA- 3´
	**R**	5´-GAAGACTCCATGCCGATCAT- 3´
**GAPDH**	**F**	5´- TTGGTTTCCACTGACTTCGTT − 3´
	**R**	5´-CTGTAGCCCCACTCGTTGT − 3´

### Data analysis

The data are displayed as the mean ± standard error (SE). For the analysis of the data, SPSS 16.0 (IBM, Armonk, New York, USA) was utilized for both one-way and multivariate analyses. The Shapiro‒Wilk normality test was used to check for normality at a significance level of 0.05. The statistical significance among treatments was determined using Tukey’s multiple comparisons test with a significance level of *P* < 0.05. The “Corrplot” software was used to analyze and identify correlations among the data from various disciplines. Using the approach described by Soetewey [66], we calculated and presented the correlation coefficients for the relationships between the variables. A distance tree was created using PAST ver. 4.02 software to compare the general responses of the eight wheat cultivars. The tree illustrates the connections between the cultivars using the UPGMA method [67]. In addition, the same program was utilized to generate a principal component analysis (PCA) scatter plot, which effectively demonstrated the degree of similarity among the cultivars based on the dice coefficient. By utilizing the R-studio interface and R software, a heatmap matrix was created to perform a multivariate analysis (R Studio Team [68]; R Core Team [69]).

## Results

### Plant growth parameters

**Fig. 1:**
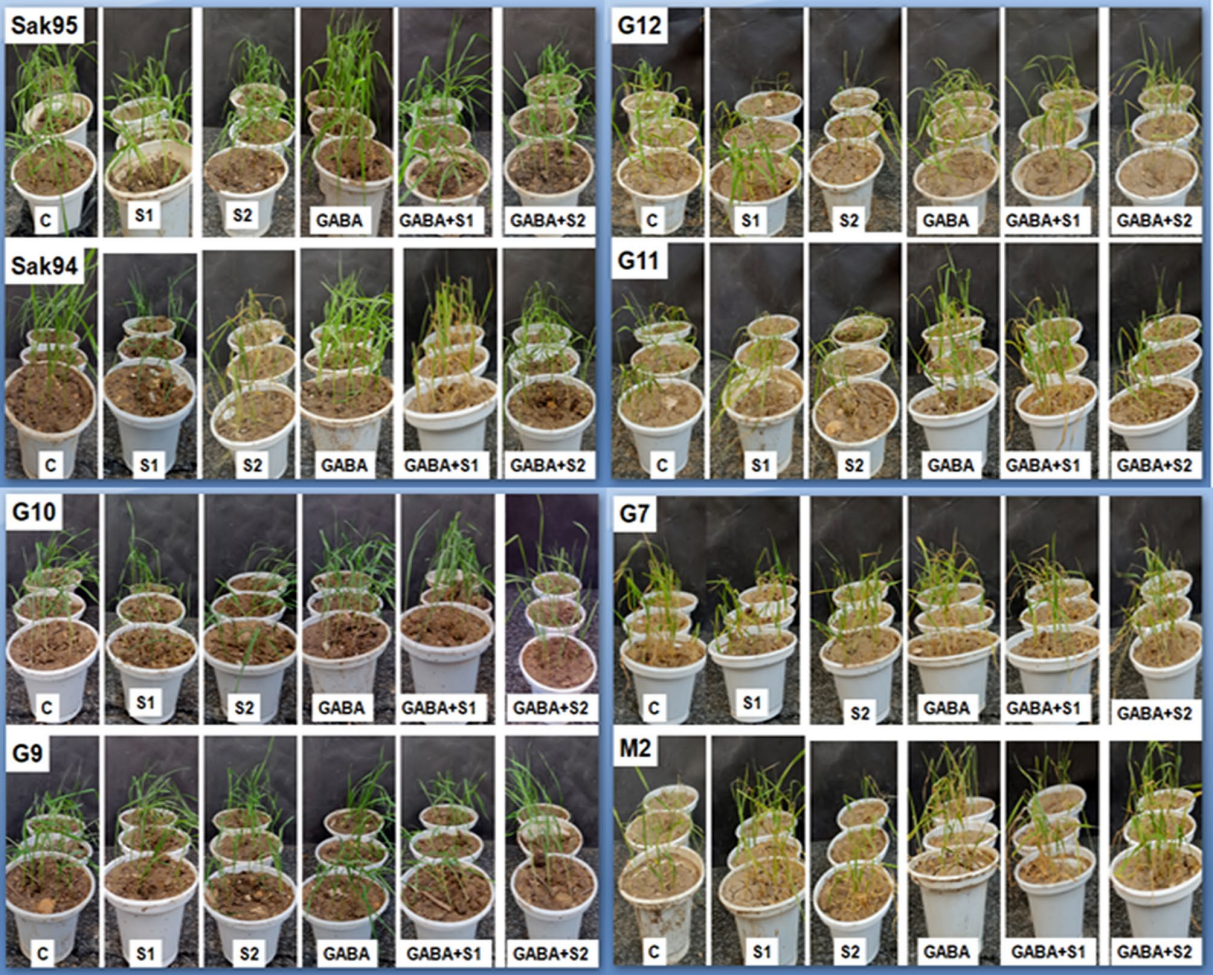
Photographs illustrating the examined wheat cultivars; M2: Misr 2, Sak94: Sakha 94, Sak95: Sakha 95, G7: Gemmiza 7, G9: Gemmiza 9, G10: Gemmiza 10, G11: Gemmiza 11, and G12: Gemmiza 12 under control, the applied salinity, and GABA treatments and changes in plant length

**Fig. 2:**
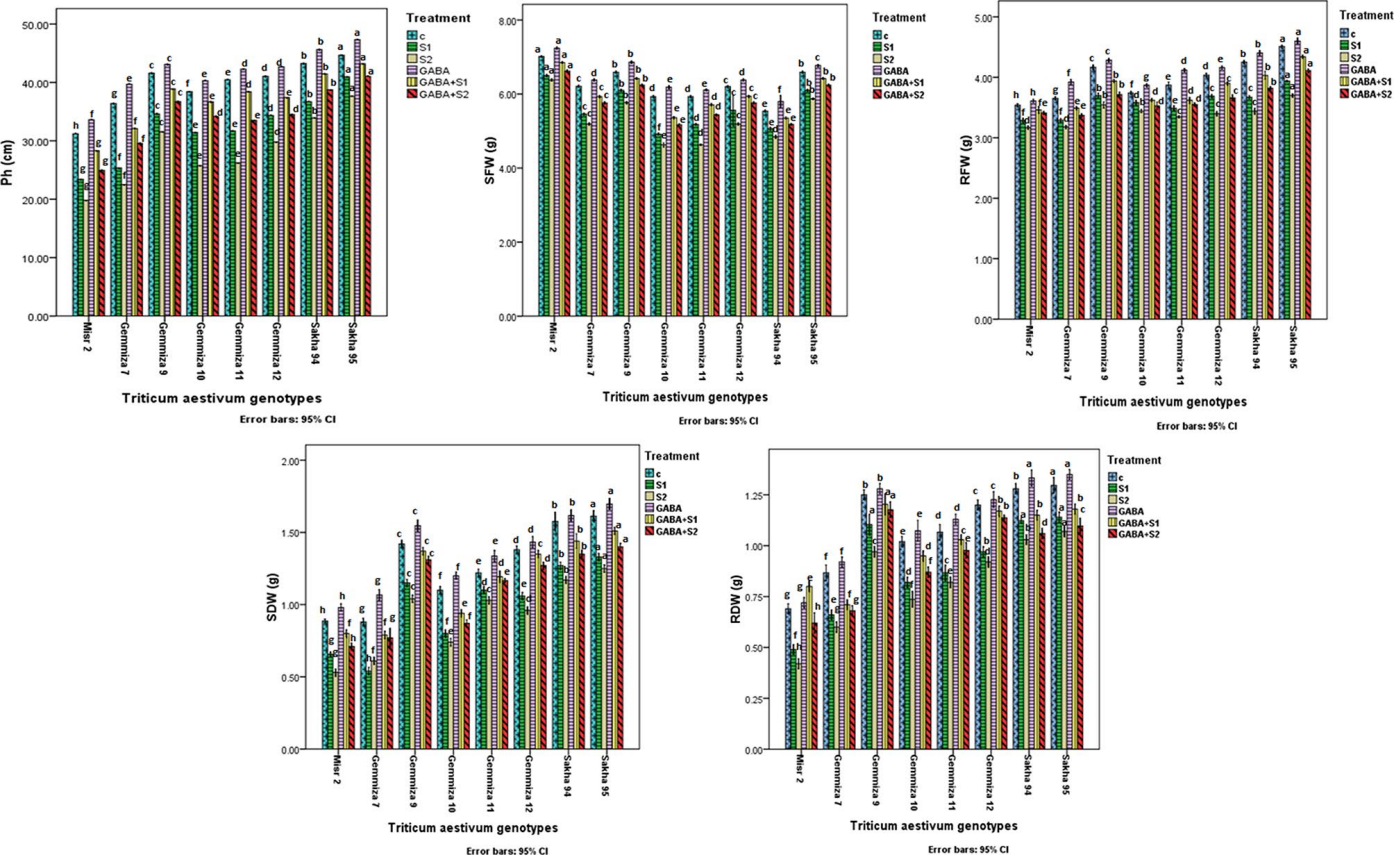
Histograms showing the variation of the examined growth parameters; plant length (Ph) in cm, shoot fresh weight (SFW) in g, shoot dry weight (SDW) in g, root fresh weight (RFW) in g, and root dry weight (RDW) in g in the examined wheat cultivars under the control, the applied salinity, and GABA treatments. Bars with different letters indicate significant differences between treatments, expressed as the mean of three replicates ± SDs

To evaluate the plant growth parameters, the plant length, shoot fresh weight (SFW), root fresh weight (RFW), shoot dry weight (SDW), and root dry weight (RDW) of the eight wheat cultivars were measured (Fig. 1). Compared to both the control group and the groups subjected to the two salinity treatments, the GABA treatment significantly improved plant growth. Notably, the salinity and GABA treatments had the least impact on CV. Sakha 95 and CV. Giza 9. Fig 2 shows the variation in the measured growth parameters of the examined wheat cultivars in the control group, where the salinity and GABA treatments were applied. By comparison, the control plants of the two Sakha cultivars were taller than those of the other six cultivars (43.22 cm for CV. Sakha 94 and 44.67 cm for CV. Sakha 95), while CV. Misr 2 was the shortest of the examined cultivars (31.22 cm). Detailed measurements are given in supplementary Table S1. GABA application at 3 mg l^− 1^ increased the plant length (Ph) of all eight wheat varieties and mitigated the reduction in plant length induced by the two salinity treatments at 8000 ppm (137 mM) and 14,000 ppm (205 mM). The most significant variation in Ph was observed between CV. Sakha 95 and CV. Misr2, followed by CV. Sakha 94 and CV. Misr2, while the lowest Ph was reported between CV. Gemmiza 9 and CV. Gemmiza 12. The changes in shoot fresh weight (SFW) are illustrated in Fig. 2 and Table S1. The changes in shoot dry weight are illustrated in Fig. 2 and Table S1. In addition, the variations in the root fresh weight (RFW) and root dry weight (RDW) are also given in Fig. 2 and Table S1, respectively. In general, the application of GABA increased the plant growth parameters compared to those of the control. However, the salinity treatments alone and combined reduced all the plant growth indices measured as plant length or fresh and/or dry biomass.

### Changes in photosynthetic pigment and gas exchange parameters

**Fig. 3:**
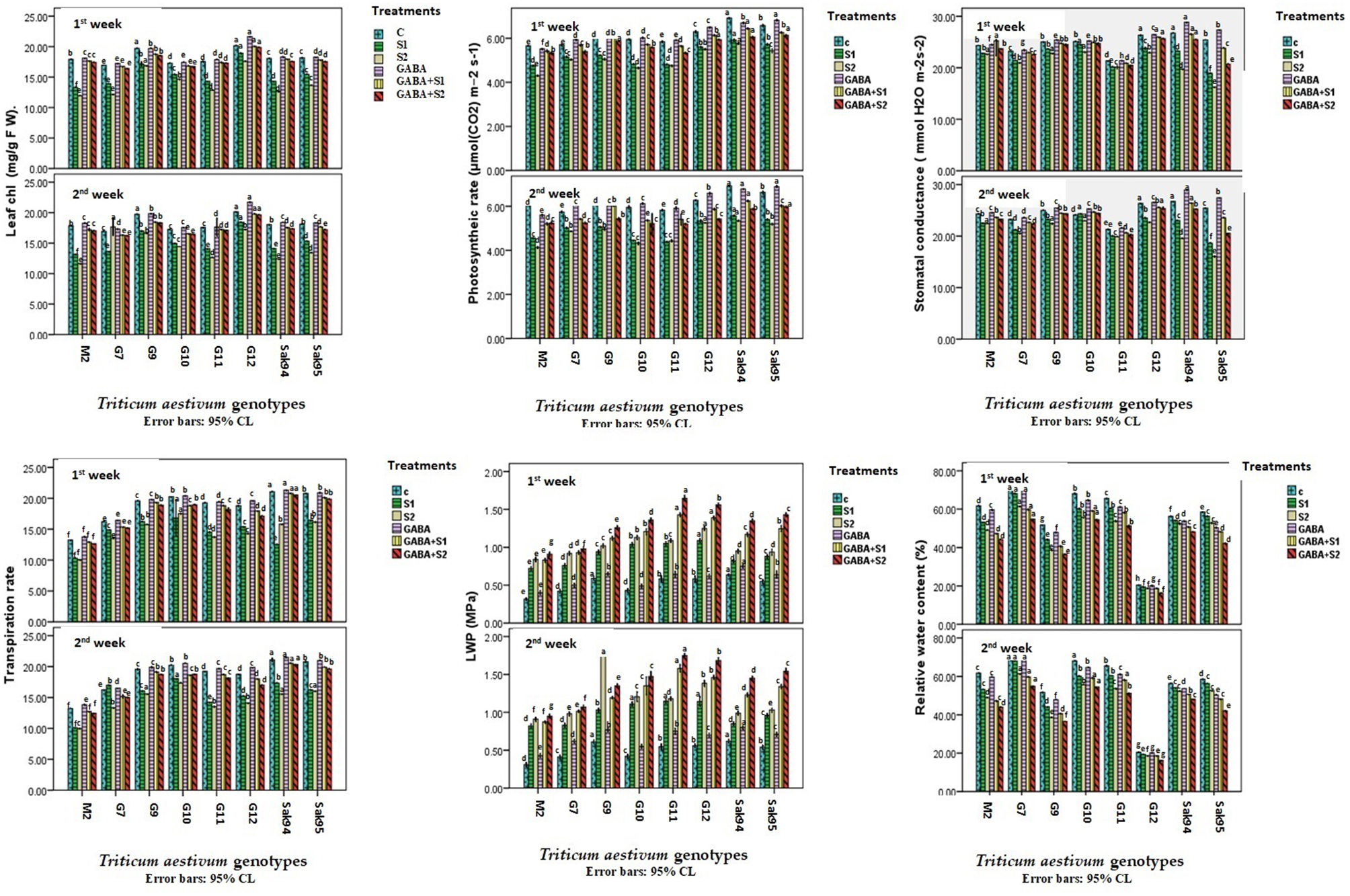
Histograms showing the variation of the leaf photosynthetic pigments (leaf chl.), photosynthesis rate, stomatal conductance, transpiration rate, leaf water potential (LWP), and relative water content (RWC) in the examined wheat cultivars under control, the applied salinity, and GABA treatments. Bars with different letters indicate significant differences between treatments, expressed as the mean of three replicates ± SDs.

In this work, several physiological parameters were measured at two different time points, one and two weeks after the salinity treatment (Fig. 3). Photosynthetic pigments (leaf chlorophyll) and the net photosynthetic rate (Pn) were measured for the control nonsalinized plants and salt-stressed plants. GABA alleviated salt stress in the plants of the examined wheat cultivars (Fig. 3 and Table S2). The results showed that CV. Gemmiza 12, followed by CV. Gemmiza 9, had the highest leaf chlorophyll content, while CV. Gemmiza 7 had the lowest leaf chlorophyll content among the control plants. Similarly, the control plants of CV. Gemmiza 7 had the highest Pn, whereas those of CV. Gemmiza 10 had the lowest Pn (Fig. 3 and Table S2). Salt stress triggered a pronounced reduction in leaf chlorophyll content (with S1 and S2 single treatments) and, consequently, a Pn rate below those detected in control wheat leaves. GABA treatment of the control plants induced a slight increase, but the change was not significant. However, compared with those in the salt-stressed samples, the greatest effects on leaf chlorophyll content and the Pn were alleviated by the GABA treatment combined with salt stress (S1 or S2) (Fig. 3 and Table S1). GABA in combination with S1 or S2 reduced the leaf chlorophyll content and Pn to a similar extent to those of the control wheat samples, especially for the Gemmiza 7, Gemmiza 9, and Gemmiza 12 cultivars.

The gas exchange parameters (Gs and Tr) were quantified and are presented in Fig. 3 and Table S2. CV. Sakha 94, followed by CV. Gemmiza 12, had the highest Gs, while CV. Gemmiza 11 had the lowest Gs among the control plants. Additionally, in the control plants, the highest Tr was recorded for CV. Sakha 94, followed by the 10 wheat cultivars Sakha 95 and Gemmiza, whereas CV. Misr 2 had the lowest Tr (Fig. 3 and Table S2). Notably, GABA application in combination with S1 or S2 salinity restored the optimum gas exchange parameters to a similar extent to those of the control wheat samples, viz., Sakha 94, Sakha 95, and, to a lesser extent, CV. Gemmiza 10.

In conclusion, CV. Gemmiza 9 exhibited increased photosynthesis-related parameters, while CV. Sakha 94 exhibited increased potential for the expression of gas exchange parameters. Additionally, the greatest impact was detected after 2 weeks of GABA treatment in combination with S1.

### Leaf relative water content and water potential

Under saline conditions, the LWP increased by at least 30% in salt-stressed plants compared with that in the corresponding controls, particularly after two weeks (Fig. 3 and Table S2). GABA had the most significant effect on the S2 saline group, especially after two weeks of exposure, when CV and Gemmiza 9 had the greatest effect. GABA addition alleviated LWP in salt-stressed samples subjected to S2 saline conditions by 185%, 200%, and 218% in CV. The LWP in the Stakha 95, CV. Gemmiza 12, and CV. Gemmiza 11 treatment groups was greater than that in the control group (Fig. 3 and Table S2). In addition, compared with those of the control plants, the salt treatments reduced the relative water content (RWC%) of all the studied wheat cultivars, except for Gemmiza 12, followed by Sakha 94 and Sakha 95 (Fig. 3 and Table S2). Compared with those in the control samples, the RWC% in the GABA-only treatment group was comparable to or less than that in the control group (Fig. 3 and Table S2). Notably, GABA application contributed to salt stress by decreasing the RWC% less than in the control and salt-stressed wheat samples (Fig. 3 and Table S2).

### Stress-induced biomarkers

**Fig. 4:**
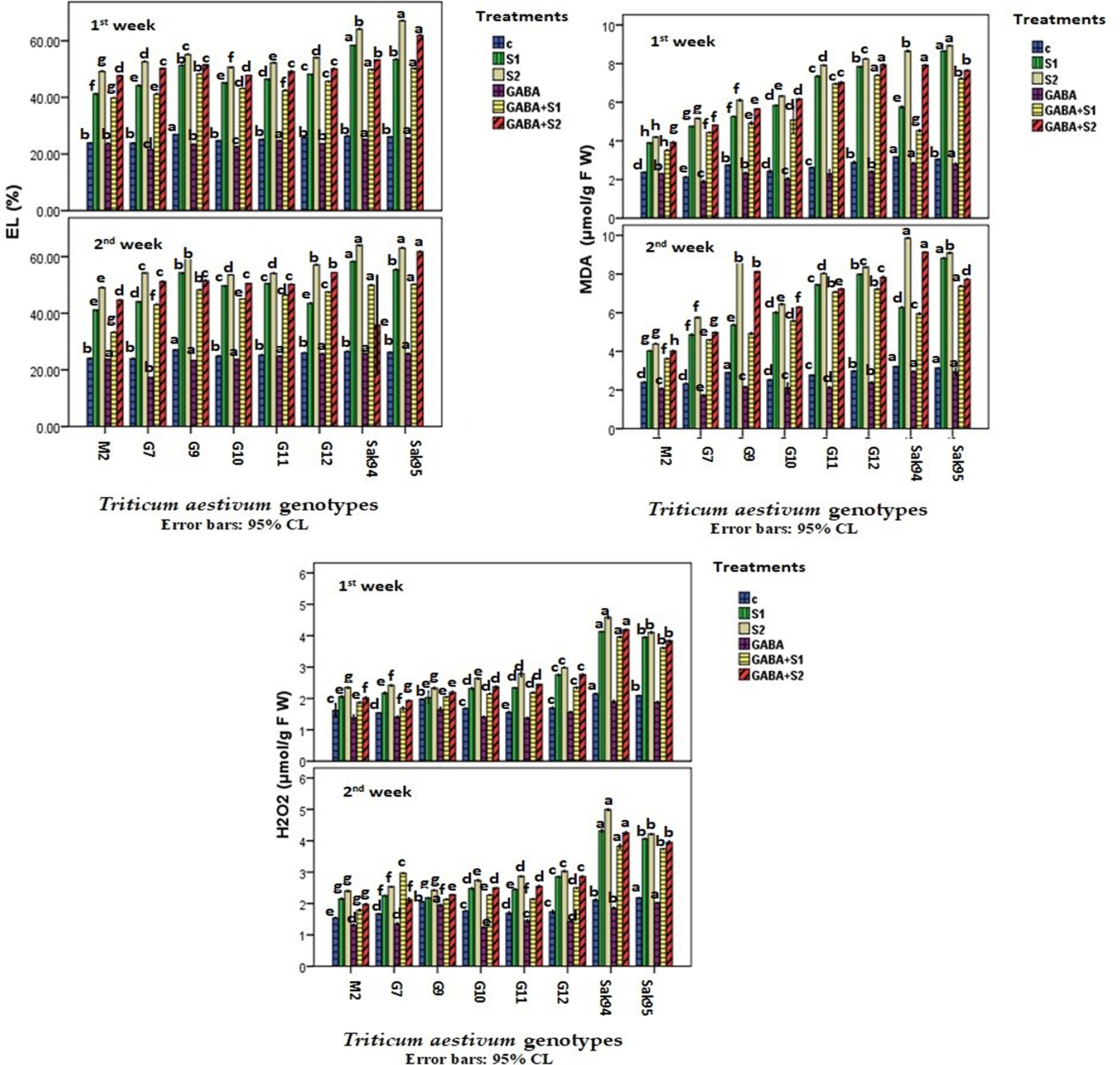
Histograms showing the variation of the stressed-induced biomarkers (hydrogen dioxide; H_2_*O*_*2*_ as µmol g -1 FW (A) and electrolyte leakage percentage; EL%) in the examined wheat cultivars under control, the applied salinity, and GABA treatments. Bars with different letters indicate significant differences between treatments, expressed as the mean of three replicates ± SDs

The changes in stress-induced biomarkers in the examined wheat cultivars under control and saline conditions in the presence or absence of GABA (individually or in combination) are presented in Fig. 4, and the detailed results are given in Table S3. The lowest H2O2 concentration was detected in the GABA-treated plants under CV. Misr 2, followed by CV. Gemmiza 7. Generally, salt stress increased the H_2_O_2_ concentration from approximately 100% in CV Misr 2 (after one week) to more than 300% in CV Sakha 94 (after 2 weeks). Similar findings were recorded following the application of S2 salt stress, in which the H_2_O_2_ concentration increased from 100% in CV Misr 2 (after one week) to more than 250% in CV Sakha 94 (after 2 weeks) (Table S3). Compared with salt treatment and the control treatment, GABA treatment alone reduced H2O2 levels. However, GABA application combined with S1 or S2 saline decreased H2O2. For example, compared with CV. Sakha 94, GABA combined with S2 salt stress caused a decrease of 16.0%, while S2 salt stress caused a decrease of 16.0% (Table S3).

Overall, the results of the monitoring of lipid peroxidation in the examined wheat cultivars under the control, applied salinity, and GABA treatments (individually or in combination) are shown in Fig. 4, and Table S3 shows that different wheat genotypes treated with GABA had the lowest MDA contents among the salt-treated groups. The minimum MDA contents were recorded in CV. Misr 2 and Gemmiza 7 plants were treated with GABA. In addition, salt stress significantly promoted the MDA content in both the S1 and S2 salt treatments. The maximum increase in the MDA content after two weeks was greater than 500% at S2 compared with that at Sakha 94, Sakha 95, and Gemmiza 12 (Fig. 4; Table S3).

Moreover, the combination of GABA and salt stress significantly decreased the MDA content compared with that in salt-stressed plants. In general, GABA alleviated the inhibitory effect of salt stress by increasing the MDA content. Similarly, control and stress-induced electrolyte leakage (El%) changes are illustrated in Fig. 4 and Table S3. The El% of the wheat cultivars Saka95 and Saka94 was greater in the S2 group than in the CV group. Gemmiza 7 and Misr 2 had the lowest El% in the salt and control groups under the GABA treatment. In this regard, the single application of salt or salt stress combined with GABA induced a significant increase in El% (Fig. 4; Table S3).

### Proline and total protein contents

**Fig. 5:**
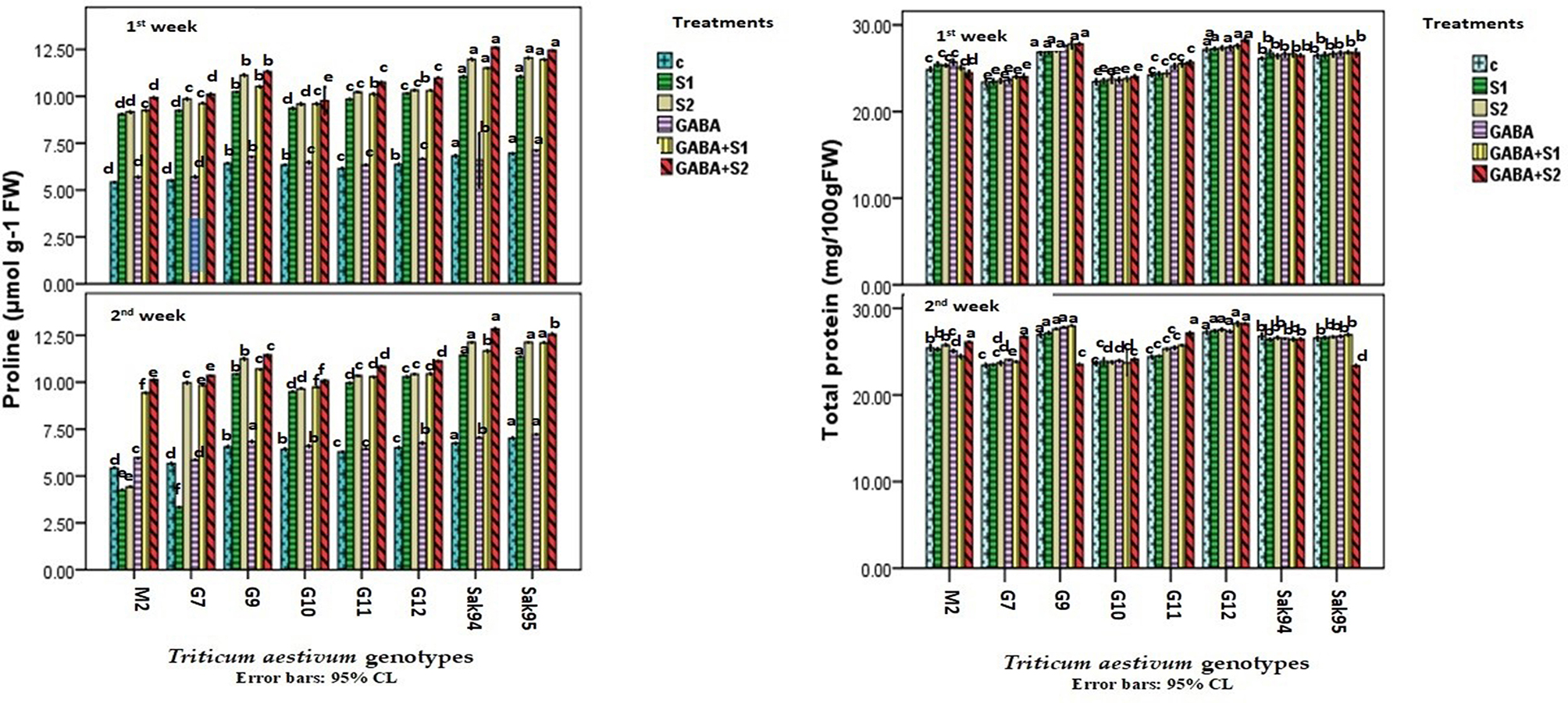
Histograms showing the variation of the proline content as µmol mg -1 FW (A) and total protein content as µmol 100 mg -1 FW (B) in the examined wheat cultivars under control, the applied salinity, and GABA treatments. Bars with different letters indicate significant differences between treatments, expressed as the mean of three replicates ± SDs

The changes in the proline content in the examined wheat cultivars under different types of applied treatments are illustrated by the histograms presented in Fig. 5 and the detailed measurements given in Table S3. The highest proline content in the control samples was recorded for CV. Sakha 95. Salt stress regimens (S4 and S2) increased the proline content. The mean percentage increase ranged from 66.78 to 69.48% in S1 and from 69.19 to 79.7% in S2, above the control value (Fig. 5 and Table S3). Overall, compared with the control treatment, the individual GABA treatments increased the proline content. However, the combination of GABA and S1 or S2 increased the proline content above that in salt-stressed plants. Although the individual salt application increased the proline content in all studied cultivars, this was not the case for CV. Misr 2, where the proline content decreased by 77% and 73.8% in the S4 and S2 treatments, respectively. Similarly, in CV. Gemmiza 7, the proline content decreased by 40.89% in S1 but increased by 76.14% in S2 compared to that in the nontreated control samples (Fig. 5 and Table S3). In addition, compared with that of the control plants, the protein content was unaffected by the salt stress treatments and/or the individual or combined application of GABA (Fig. 5 and Table S3). Notably, the GABA + S2 treatment reduced the protein content in CV. Compared with the control and salt stress treatments, the addition of Sakha 95 increased the protein content in CV. Misr 2 (Fig. 5 and Table S3).

### Antioxidant enzymes

**Fig. 6:**
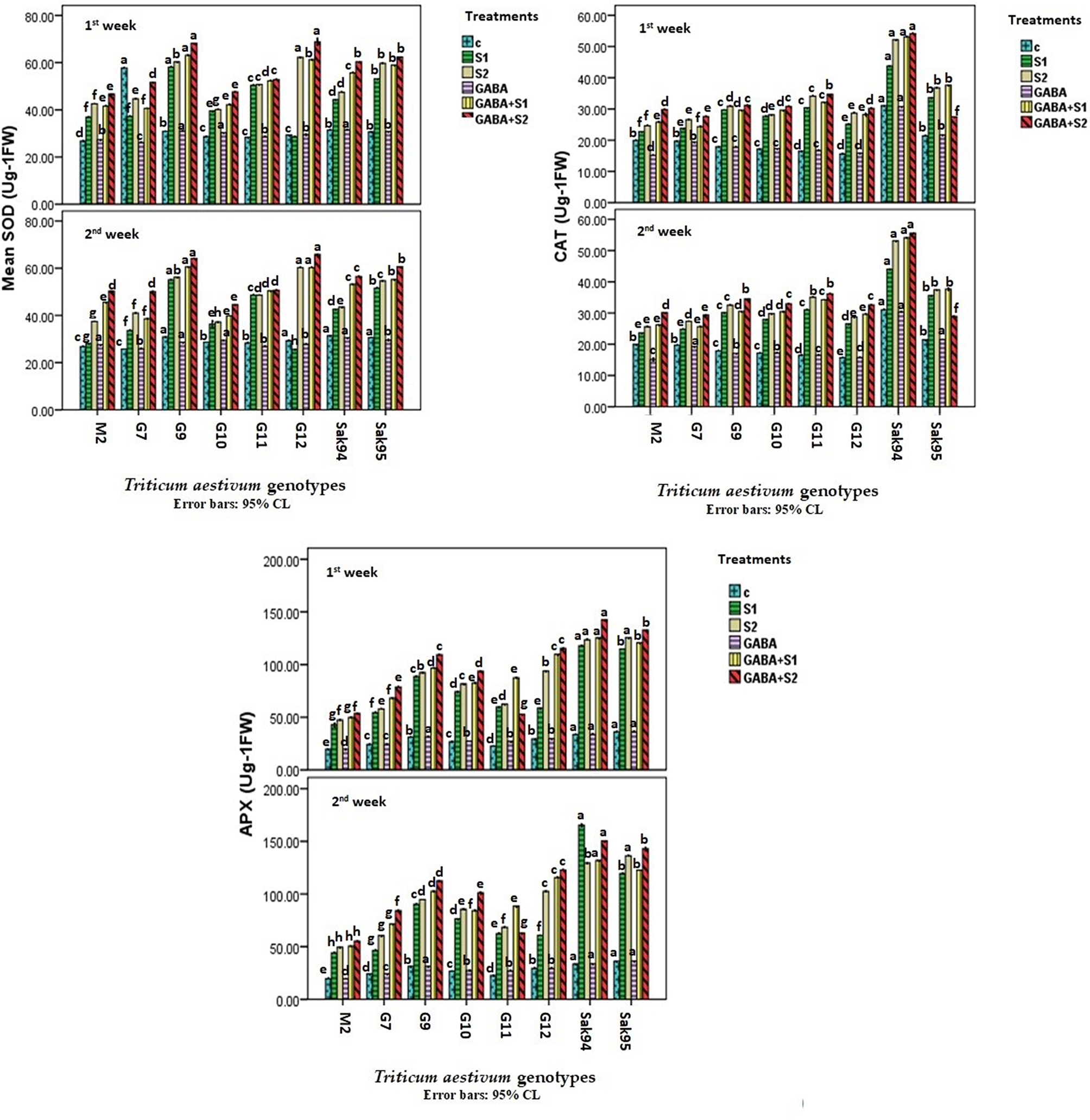
Histograms showing the variation of the antioxidant’s enzymes SOD (Ug -1 FW) and CAT (Ug -1 FW) in the examined wheat cultivars under control, the applied salinity, and GABA treatments. Bars with different letters indicate significant differences between treatments, expressed as the mean of three replicates ± SDs

The fluctuations in antioxidant enzyme (SOD, CAT, and APX) activities were investigated for nontreated and salt-stressed wheat cultivars treated with GABA, and their interactions are presented in Fig. 6 and Table S3. Compared with those of the control samples, all the applied salt treatments significantly increased SOD activity. Additionally, compared with the control treatment, GABA treatment alone triggered an increase in SOD activity, which was not significant (Fig. 6 and Table S3). However, SOD activity was significantly induced in combination with the S4 and S2 treatments in the viz., Sakha 95, Gemmiza 9, and Gemmiza 12 cultivars. In addition, the highest CAT activity in the control nontreated samples was recorded for CV. Sakha 94. The individual GABA applied to the control samples notably reduced the CAT activity (Fig. 6 and Table S3). All the applied salt treatments, alone or combined with GABA, increased the CAT activity in a similar manner (Fig. 6 and Table S3). Notably, the combination of GABA and salt treatments triggered the greatest increase in CAT activity in CV. Sakha 94. The results showed that both the Sakha 94 and Gemmiza 9 cultivars exhibited maximum APX activity in the control nontreated samples (Fig. 6 and Table S3). Individual salt stress (S4 and S2) induced greater APX activity than that recorded for the control nontreated samples. Tremendous increases in APX activity were detected in CV. Sakha 94 and CV. Sakha 95 by 252% and 230.38% (in the case of S1) and by 230.38% and 276.86% (in the case of S2), respectively, after two weeks (Fig. 6 and Table S3). In this regard, APX activity exhibited pronounced fluctuations of 106.7% (in the case of S1) and 249% (in the case of S2) in CV. Gemmiza 12.

**Fig. 7:**
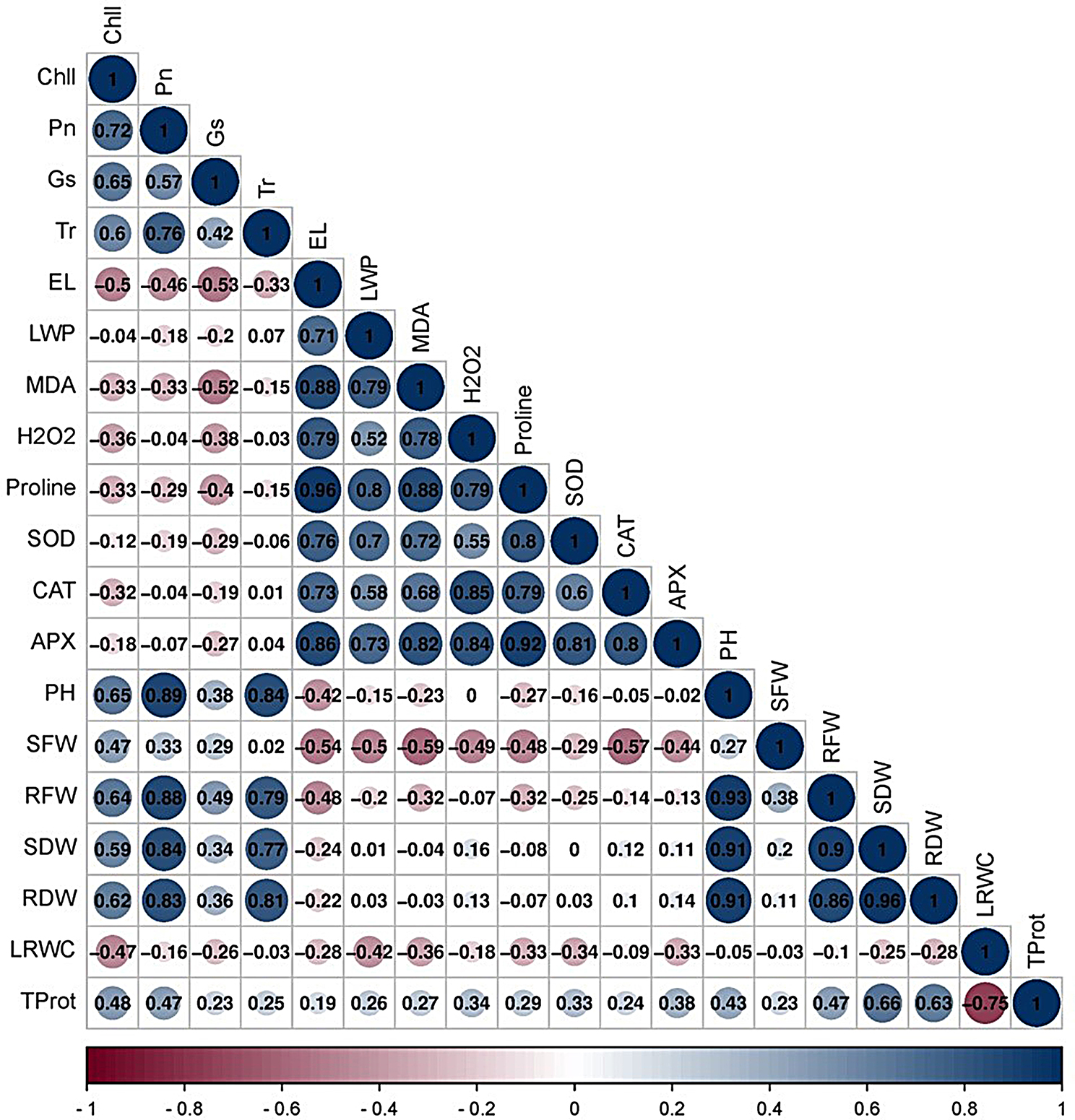
Correlogram based on the correlation coefficients of morphological and metabolic measurements. The blue colour indicates the positive correlation between measurements, while the red colour assumes the negative one

**Fig. 8:**
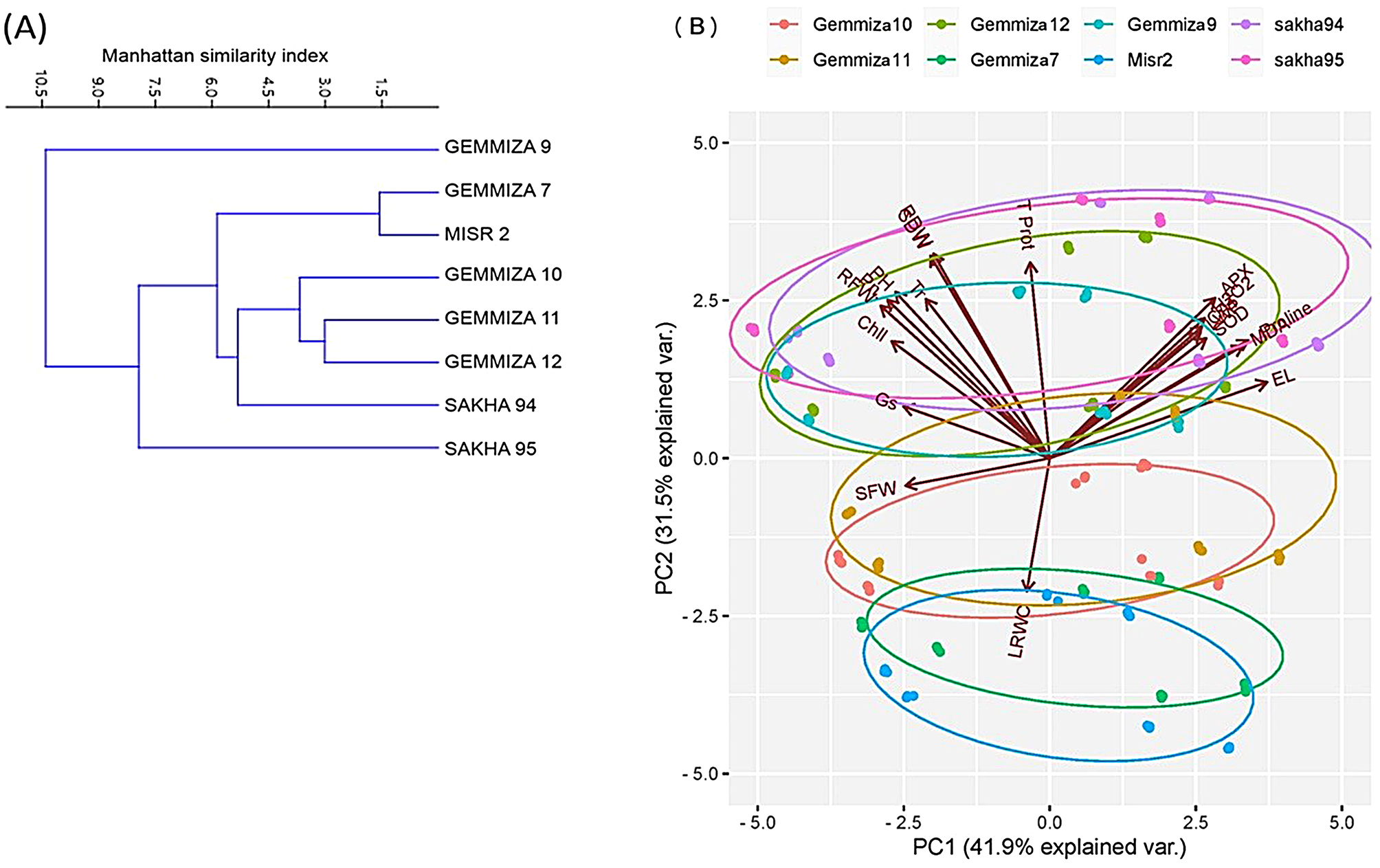
UPMGA hierarchical clustering (**A**) and PCA scatter diagram (**B**) analyses based on the data outcomes of morphological and metabolic attributes of the studied eight wheat cultivars under the applied salinity and GABA treatments

Further conclusions were drawn by evaluating the correlations between the morphological, physiological, and phytobiochemical variables for the eight examined wheat cultivars. The results are shown in Fig. 7. Fig 7 presents a Pearson correlation matrix of the morphological, phyto-biochemical, and enzyme activity data. The most notable correlation found was a strong positive correlation of 0.96 between proline and the SOD enzyme, as well as between the relative water content and root dry weight. There was a strong correlation of 0.93 between root fresh weight and shoot dry weight. There was a slight positive correlation of 0.01 between the APX and CAT antioxidant enzymes, as well as between the shoot dry weight and root dry weight. A slight negative correlation of -0.03 was detected between the dry weight of the roots and the relative water content. A plot was created to examine the connections between the physical and chemical traits of different wheat cultivars and their enzyme activity. The size of the circles reflects the strength of the correlations, with larger circles indicating stronger associations. The color scale is determined by the variation in the measurements that are presented. UPMGA hierarchical clustering was used to represent the salinity tolerance response indices of the vegetative, physiological, and biochemical responses measured for all the cultivars. Figure 8A clearly shows that CV. Gemmiza 9 stands out from the other cultivars. CV. Sakha 95 was also separated as a single branch from the other six cultivars and grouped into two clusters. One cluster included CV. Misr 2 and Gemmiza 7, while the other cluster included CV. Gemmiza 10, Gemmiza 11, and CV. Gemmiza 12. All the other wheat cultivars included in the first group were studied, with the exception of CV (Fig. 8A).

There is a clear distinction between CV. Gemmiza 9 and CV. Sakha 95, as well as other cultivars that can be grouped together. This is shown for components 1 and 2 (Fig. 8A). Principal component analysis (PCA) was conducted on the morphological and biochemical traits of various wheat genotypes to assess the grouping and relationships between the studied genotypes under different levels of salt stress. The data in Fig. 8B indicate that PCA1 explained 41.9% of the variation, while PCA2 explained 31.5%. Through principal component analysis (PCA), a clear separation of wheat genotypes was observed. Specifically, a cluster consisting of four genotypes (SAKHA 94, SAKHA 95, GEMMIZA 9, and GEMMIZA 12) was identified in the upper quadrant of the figure. The root dry weight, shoot dry weight, plant length, and APX and SOD activities are crucial factors in this differentiation. The size of the arrow represents the intensity of the variable, while the orientation of the arrow indicates the highest value of the variable. Four different genotypes (GEMMIZA 10, GEMMIZA 11, GEMMIZA 7, and Misr2) were classified in the lower quadrant using various criteria, such as relative water content, shoot fresh weight, and electrolyte leakage. This indicates that they belong to overlapping groups.

#### Gene expression analysis

**Fig. 9:**
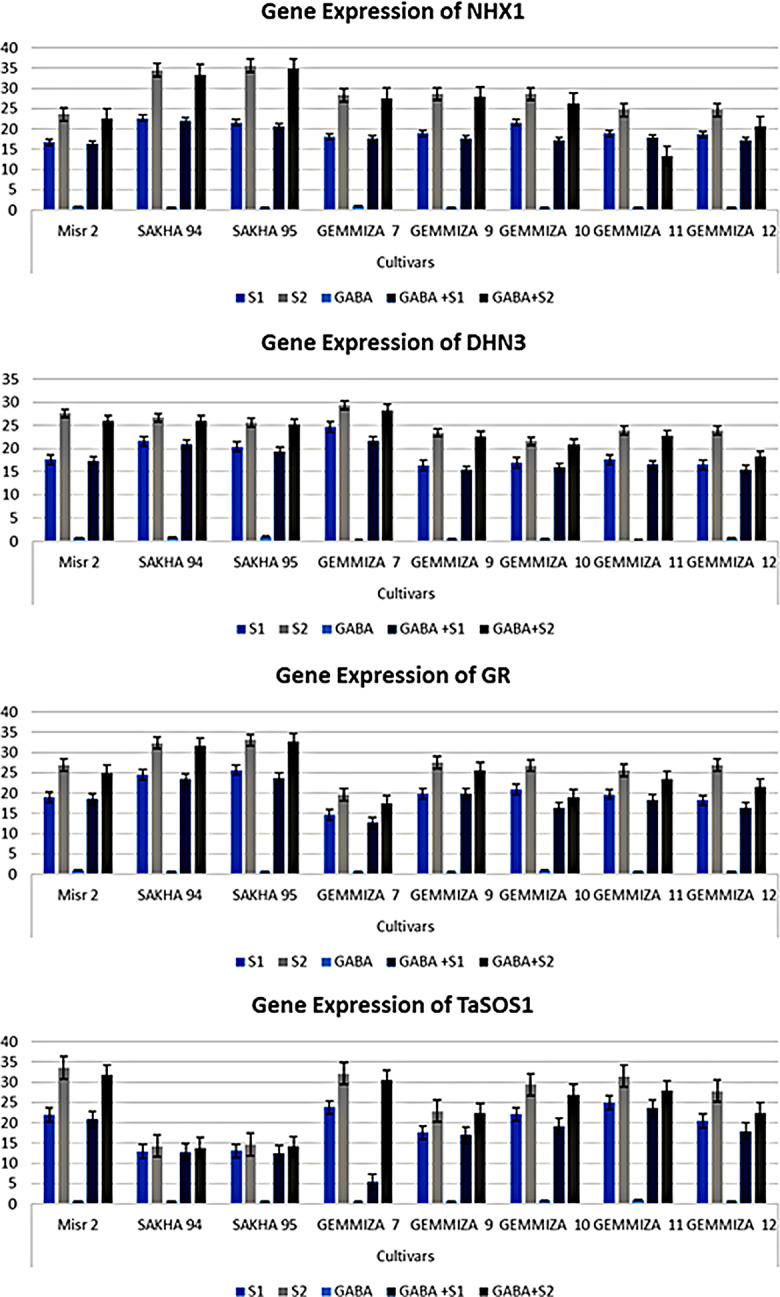
Fold change of NXH1, DHN3, GR, and TaSOS1 genes of the eight studied wheat cultivars as revealed by qRT-PCR analysis. The investigated analysis of calculated fold change of differentially expressed NXH1, DHN3, GR, and TaSOS1 gene transcripts shown in detail in Table S4. The data was analyzed by Microsoft Excel-enabled XLSTAT Version 2014.4.06. Transcriptional analysis of expressed genes was represented as fold changes compared to the relevant control sample. X axis refers to names of wheat cultivars, while the y axis refers to calculated fold change

**Fig. 10:**
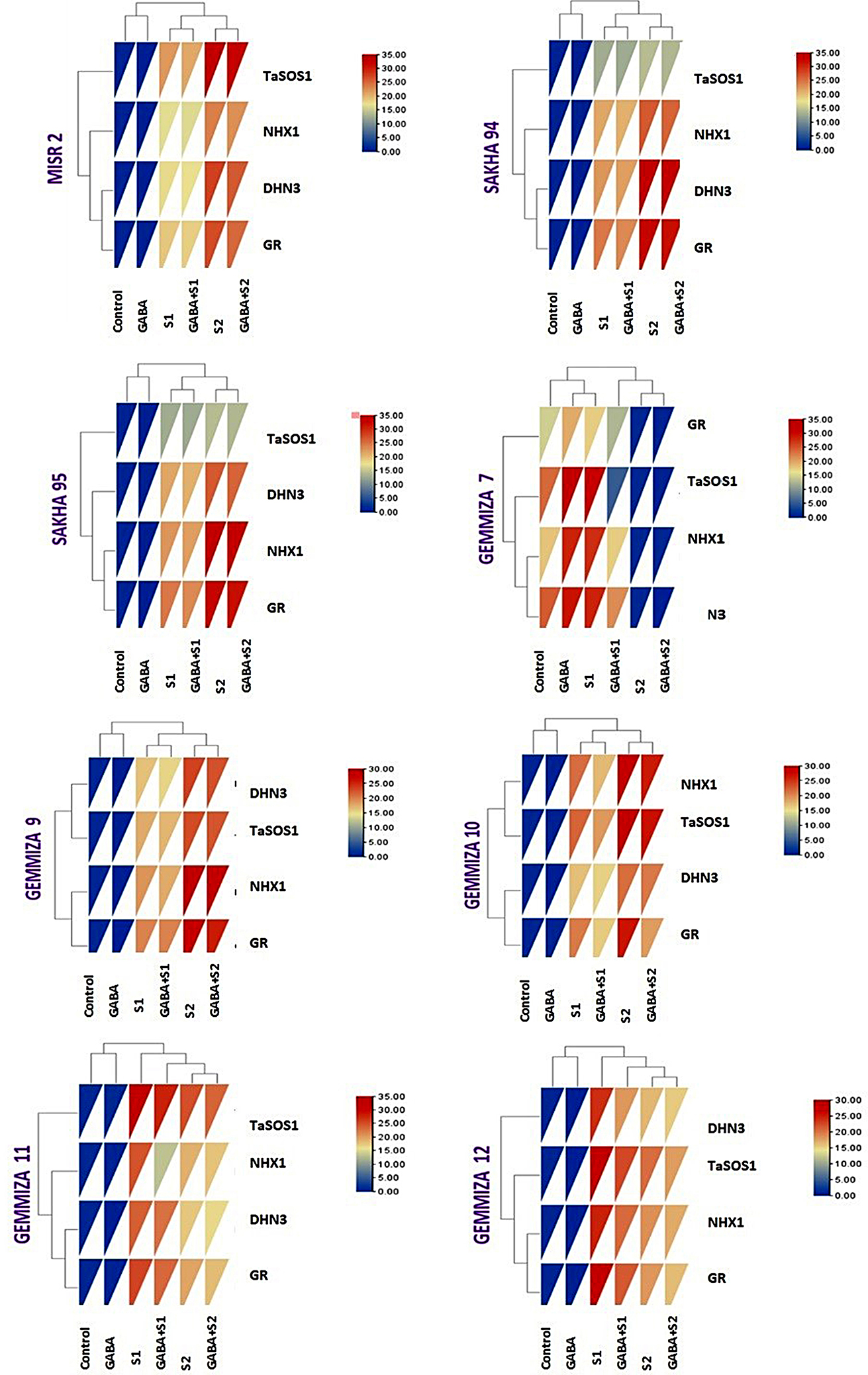
Gene expression analysis of NXH1, DHN3, GR, and TaSOS1 genes as revealed by multivariate heatmap analysis using R software

The gene expression profiles of NHX1 in response to salinity stress in the eight wheat cultivars were recorded via qRT‒PCR. All gene transcripts are presented as the fold change increase/decrease compared to control nontreated samples (Fig. 9; Table S4). In the case of the applied S1 regime, NHX1 gene transcripts were more highly expressed in CV. Sakha 94, followed by CV. Sakha 95 and CV. Gemmiza 10, with a 21.64-fold increase (upregulation) compared to that in the controls. On the other hand, the expression of NHX1 in Sakha 95 was 0.28-fold greater than that in Sakha 94 under salt stress in S2 (Fig. 9). In all the cultivars, the gene transcripts of NXH1 were upregulated by a single application of GABA, ranging from 0.64- to 0.98-fold (Table S4). The results of the combination of GABA application and salt stress were consistent with the expression profiles of NHX1 revealed by salt stress, except in CV. Gemmiza 12, where NHX1 was upregulated in the S2 treatment group by an increase in the number of genes whose expression decreased after salt stress compared with that in the control group (Fig. 9). On the other hand, the expression profile of DNH3 was greatest in CV. Gemmiza 7, followed by CV. Sakha 94 and then CV. Sakha 95, with 24.69-, 21.59-, and 20.41-fold changes, respectively (Fig. 9), following S1 application. The results of the combination of GABA application and salt stress were consistent with the expression profiles of DNH3 revealed by salt stress. The expression of GR-related genes significantly increased in CV. Sakha 95, followed by CV. Sakha 94, after salt stress application (Fig. 9, Table S4). Similarly, as reported for NHX1 and DNH3, the changes in the expression of GRs induced by the combination of GABA and DNH3 were consistent with the changes in the salt-stressed plants. The expression of the GR gene was upregulated in CV. Sakha 94 and CV. Sakha 95 because of the addition of salt stress to S2 (Fig. 9, Table S4). Similarly, the combination of GABA application with S2 maintained high GR expression in the same cultivar. GABA gene transcripts of TaSOS were more highly expressed in CV. Gemmiza 7 than in CV. Gemmiza 11, with 32.15- and 31.54-fold greater expression, respectively, in salt-stressed samples (Fig. 9). To verify the previous findings, a heatmap-based multivariate analysis of the gene expression of the studied genes was performed based on a color-coded matrix (Fig. 10). Additionally, a positive correlation was detected between NHX1, DNH3, and GR, which are differentially expressed genes, either under salt stress conditions alone (S4 and S2) or in combination with GABA treatment, especially in the Misr 2, Sakha 94, and Sakha 95 cultivars (Figs. 9 and 10). In conclusion, the most highly expressed genes among all studied genes were CV. Misr 2, CV. Sakha 94, and CV. Sakha 95, except for CV. Gemmiza 7 and CV. Gemmiza 11, which exhibited the greatest expression of the TaSOS1 gene (Fig. 9).

## Discussion

The eight wheat varieties were subjected to salinity stress and evaluated for their ability to adapt to GABA. According to the research conducted by Zhang et al. [70], Negrão et al. [71], and Al-Ashkar et al. [72], salt stress has a significant impact on plants. This stress hampers or even halts important cellular activities, ultimately resulting in a reduction in the overall dry matter of the plants. Research conducted by Soltabayeva et al. [73] revealed that plants require a significant amount of energy to cope with salt stress rather than prioritizing growth or cellular development. Thus, plants have developed sophisticated regulatory mechanisms and well-coordinated signaling cascades to cope with challenging environmental conditions. In addition, plants possess a variety of antioxidant defense mechanisms and osmoprotectants, such as GABA, to combat unfavorable environmental conditions, such as salt stress [74]. The activation of glutamic acid decarboxylase (GAD) connects GABA to signals of environmental stress, indicating the intensity and duration of that stress. In a study conducted by Ma et al. [49], GABA was shown to play a role in intracellular Ca^2+^ signal transduction pathways and the physiological responses associated with ethylene stress. Research conducted by Shelp et al. [51] revealed that GABA has growth-promoting effects on dry matter and plays a role in plant development. GABA has been shown to mitigate the negative effects of salinity stress on the morphological traits of the wheat cultivars examined. In line with these discoveries, Hassanein et al. [75] investigated the negative impacts of salt stress on the physical characteristics of coriander and the biostimulators that alleviated these effects. According to the findings of Ma et al. [49], an important factor in the series of events that starts with the detection of environmental stimuli and concludes with appropriate physiological responses could be the build-up of GABA in distressed tissue. Studying physiological features related to stress tolerance could greatly enhance wheat adaptation to unfavorable conditions, making it a valuable selection criterion. Research has shown that plants can experience cross-talk between different physiological reactions when faced with drought stress and salinity stress. For example, studies have demonstrated a relationship between high relative water content (RWC) and leaf water potential (LWP) in crops [76, 77].

Plant cells possess robust antioxidant defenses, enabling them to better withstand high concentrations of hydrogen peroxide; consequently, monitoring these levels aids in regulating fluctuations in stress-related markers. Apel and Hirt [25] reported that through a multistep monovalent reduction process, oxygen can be activated, leading to the formation of different radicals, including superoxide radicals (O^2•−^), hydrogen peroxide (H_2_O_2_), hydroxyl radicals (^•^OH), and ultimately water. In line with earlier studies, this research demonstrated that GABA has the potential to mitigate the harmful effects of salinity stress, osmotic stress, and a combination of both on rice [78]. Research on oxidative stress and redox signaling has typically involved the measurement of malondialdehyde (MDA), a marker of lipid peroxidation. This is particularly evident in studies that investigate the ways in which plants react to living and nonliving factors [57]. Our findings revealed an increase in MDA levels under salt stress conditions; Hassanein et al. [75] and Sheteiwy et al. [78] reported similar findings.

Research by Mittler et al. [22] and Szymańska et al. [79] has shown that plants possess a defense system against oxidative damage caused by reactive oxygen species (ROS). This defense system utilizes nonenzymatic antioxidants, such as proline, to protect plant cells. Notably, this study revealed that the application of GABA had a significant impact on mitigating the negative effects of salinity stress on wheat plants. Additionally, GABA treatment increased the levels of antioxidant defense elements in wheat cultivars, regardless of the severity of the stress conditions [23, 75]. The proline content increased when salt stress and GABA were applied simultaneously. Tomatoes grown in controlled environments exhibited higher levels of GABA prior to an increase in salt-tolerant soluble sugars and proline [80]. Hassanein et al. [75] demonstrated that the proline content tends to increase under salt stress conditions. Interestingly, our own findings align with this observation, particularly when proline is combined with biostimulators. Proline is a crucial molecule that tends to accumulate in response to stressful conditions. Previous research [81] has shown that proline plays a vital role in stress tolerance and is produced in tolerant transformants at high temperatures.

According to Mishra et al. [77], the assessment of antioxidant enzymes is vital for neutralizing harmful reactions caused by reactive oxygen species (ROS). It can be used as a biochemical early indicator for selecting genotypes that show strong antioxidant activity and possibly high yield. When faced with oxidative stress from an increase in reactive oxygen species (ROS) and malondialdehyde (MDA) levels, wheat responds by increasing the production of antioxidant enzymes such as catalase and phosphodiesterase (APX) [82]. In wheat plants subjected to salt stress, researchers discovered that certain enzymes, including catalase, superoxide dismutase, and apolipoprotein X, exhibited increased activity. This increase in activity is believed to be a response to the accumulation of reactive oxygen species (ROS) caused by hydrogen peroxide and superoxide [83]. According to Ahanger et al. [84], a method to safeguard wheat from the harmful effects of salt-induced oxidative stress is by boosting the production of antioxidants, osmolytes, and secondary metabolites. There is a correlation between reduced chlorophyll and photosynthetic activity and the production of enzymatic antioxidants such as SOD, CAT, and APX [75, 85]. The results of this study support the findings of previous research conducted by Soliman et al. [86] and Abd-Ellatif et al. [87] regarding the increased activity of antioxidant enzymes in protecting against the harmful effects of reactive oxygen species (ROS). The permeability of plasma and the state of the cell wall can be influenced by abiotic stressors such as salinity and drought. These stressors can have an impact on electrolyte leakage, relative water content, and other leaf water potentials. Through transcriptome analysis, Duarte-Delgado et al. [45] discovered that during the osmotic phase, the tolerant wheat genotype activates genes involved in cell wall synthesis and calcium binding. In addition, it was suggested that these steps could have a significant impact, as they contribute to more stable photosynthesis under enhanced salt stress [88, 89].

In addition, it was believed that the tolerant genotypes are affected by the targeted activation of specific Na^+^/Ca^2+^ exchangers and ABC transporters, indicating that these genes play a role in the salt tolerance of wheat by excluding sodium. Several factors could contribute to this phenomenon, such as genetic pathways that prevent the entry of harmful salt ions into cells or the accumulation of these ions in subcellular organelles. These mechanisms may help protect plants from damage during the germination process. In addition to its potential signaling role, GABA is widely recognized for its role as a metabolite in the trimethylamine (TCA) cycle and in carbon and nitrogen metabolism [90]. A recent study revealed that when medicinal *Andrographis paniculata* plants are exposed to exogenous GABA, the transcription of two nitrate transporter genes (NRT2.4 and NRT3.2) increases, as shown by recent research using 15 N isotopic tracing. Under conditions of nitrogen scarcity, plants can increase their uptake of NO_3_^−^, as demonstrated by previous studies [91, 92]. Past studies have examined the impact of salt stress on MDA levels and the influence of external GABA. Plants that received a boost in GABA showed a decrease in malondialdehyde and hydrogen peroxide levels when they were exposed to salt, unlike plants that were under stress and did not have GABA. Under salt stress, wheat leaves exhibited elevated levels of MDA and H_2_O_2_. Interestingly, the addition of GABA did not lead to a reduction in these levels.

Based on our research, it appears that the eight wheat cultivars we studied have varying levels of tolerance to salt stress. This is evident from the significant expression of genes such as NHX1, DHN3, GR, and TaSOS1 under salt stress. The expression levels of the NHX1, DHN3, GR, and TaSOS1 genes were greater in salt-treated seedlings than in untreated seedlings but lower in salt-treated seedlings than in control seedlings. Plants have developed mechanisms to safeguard themselves during germination and early growth against harmful salt ions (Na^+^ and Cl^-^) [93]. This protection can be attributed to genetic pathways that prevent these ions from entering cells or by storing them in subcellular organelles [76]. Under salt stress, the expression of various genes, such as NHX1, DHN3, GR, and TaSOS1, increased. Furthermore, of the eight wheat cultivars analyzed via qRT‒PCR, CV. Misr 2, CV. Sakha 94, and CV. Sakha 95 displayed the most pronounced variations in the expression levels of the NHX1, DHN3, and GR genes. Interestingly, the TaSOS1 gene was most strongly expressed in CV. Gemmiza 7 and CV. Gemmiza 11. The development and yield components of wheat are adversely impacted by salt stress, but these effects are alleviated by GABA treatment.

There was a clear distinction between CV. Gemmiza 9 and CV. Sakha 95, as well as between other cultivars that can be grouped based on components 1 and 2 (Fig. 8A). Principal component analysis (PCA) was conducted on the morphological and biochemical traits of various wheat genotypes to assess the grouping and relationships between the studied genotypes under different salt stress conditions [15, 23]. According to Fig. 8B, PCA1 explained 41.9% of the variation, while PCA2 explained 31.5%. Through principal component analysis (PCA), a clear separation of wheat genotypes was observed. Specifically, a cluster consisting of four genotypes (SAKHA 94, SAKHA 95, GEMMIZA 9, and GEMMIZA 12) was identified in the upper quadrant of the figure. The variables of root dry weight shoot dry weight, plant length, APX, and SOD enzymes are crucial factors in this differentiation. The size of the arrow represents the intensity of the variable, while the orientation of the arrow indicates the highest value of the variable. Four different genotypes (GEMMIZA 10, GEMMIZA 11, GEMMIZA 7, and Misr2) were classified in the lower quadrant using factors such as relative water content, shoot fresh weight, and electrolyte leakage. This indicates that they belong to overlapping groups.

## Conclusions

The global issue of soil salinization must be addressed through the implementation of several research initiatives aimed at enhancing and optimizing crop productivity. Salinity stress adversely impacts the development, biomass, and physiological metabolism of most crop plants, leading to a significant reduction in output. GABA functions as a crucial molecule that serves as a central connection for several pathways impacted by salt stress. It also offers an alternative metabolic pathway known as the GABA shunt, which enhances energy production and mitigates the harmful consequences of salt. The present work demonstrated that the application of exogenous GABA to the leaves of wheat plants plays a crucial role in enhancing growth, enhancing physiological and chemical characteristics, and regulating tolerance to salinity stress. These findings provide insight into how the application of GABA impacts the examined wheat types by lowering the salinity stress caused by reactive oxygen species (ROS) and influencing the expression of salt tolerance-related transcription factor genes. More precisely, it can enhance growth and metabolic processes and reduce the levels of H_2_O_2_ and MDA by increasing the activity of antioxidant enzymes. This can result in improved photosynthetic biosynthesis and regulation of stomatal conductance, ultimately leading to increased wheat yield in the presence of salt stress. The reaction of plants to salinity stress involves a network of interconnected physiological, biochemical, and molecular mechanisms. The foliar application of GABA has shown agronomic importance because of its potential for managing salt-sensitive crops in soils affected by salinity. It enhances plant resistance to salt stress, thereby improving current methods for mitigating the effects of salt stress and promoting plant growth under harsh conditions. Additionally, GABA plays a role in plant conservation and breeding.

### Electronic supplementary material

Below is the link to the electronic supplementary material.

Supplementary Material 1

## Data Availability

All recorded, measured, and analyzed datasets (e.g., independent measurements of the three biological and technical replicates) were completely incorporated within the manuscript’s main text and its accompanied supplementary data files.
