# Supplementary material for: Ameliorative impacts of gamma-aminobutyric acid (GABA) on seedling growth, physiological biomarkers, and gene expression in eight wheat (Triticum aestivum L.) cultivars under salt stress
Source: BMC Plant Biol. 2024 Jun 26;24:605. doi: 10.1186/s12870-024-05264-5 (PMC11201109; doi:10.1186/s12870-024-05264-5)
Supplement: Supplementary file 1 — Supplementary Material 1 [file 12870_2024_5264_MOESM1_ESM.doc]

**Table S1:** Variation of the examined growth parameters (Plant height, shoot fresh weight, shoot dry weight, root fresh weight, root dry weight) in the examined wheat cultivars under different NaCl concentrations alone or with addition of GABA treatments. Means ± SEs, *n* = 4, at *P* < 0.05 according to Duncan’s test.

| **Wheat cultivars** | **Mean ±SD** | **Control** | **S1** | **S2** | **GABA** | **GABA +S1** | **GABA+S2** |
| --- | --- | --- | --- | --- | --- | --- | --- |
| **Misr 2** | Ph (cm) | 31±0.015 | 23.41±0.01 | 19.81±0.01 | 33.61±0.01 | 28.31±0.01 | 24.95±0.11 |
|  | SFW (g) | 7.01±0.006 | 6.5±0.01 | 6.38±0.011 | 7.24±0.01 | 6.85±0.01 | 6.62±0.01 |
|  | RFW (g) | 3.54±0.01 | 3.29±0.016 | 3.17±0.015 | 3.61±0.01 | 3.46±0.02 | 3.41±0.01 |
|  | SDW (g) | 0.89±0.006 | 0.66±0.006 | 0.53±0.01 | 0.98±0.01 | 0.8±0.01 | 0.71±0.01 |
|  | RDW (g) | 0.69±0.01 | 0.49±0.01 | 0.42±0.01 | 0.72±0.01 | 0.8±0.01 | 0.62±0.02 |
|  |  |  |  |  |  |  |  |
| **SAKHA 94** | Ph (cm) | 43.23±0.025 | 36.71±0.01 | 33.91±0.11 | 45.62±0.021 | 41.47±0.01 | 38.74±0.011 |
|  | SFW (g) | 5.54±0.01 | 5.07±0.016 | 4.84±0.015 | 5.8±0.07 | 5.35±0.01 | 5.18±0.012 |
|  | RFW (g) | 4.25±0.01 | 3.67±0.01 | 3.44±0.02 | 4.4±0.016 | 4.04±0.026 | 3.82±0.01 |
|  | SDW (g) | 1.58±0.026 | 1.27±0.01 | 1.17±0.01 | 1.62±0.016 | 1.44±0.02 | 1.35±0.01 |
|  | RDW (g) | 1.28±0.01 | 1.12±0.06 | 1.03±0.01 | 1.33±0.016 | 1.15±0.01 | 1.06±0.01 |
| **SAKHA 95** |  |  |  |  |  |  |  |
|  | Ph (cm) | 44.67±0.01 | 40.95±0.11 | 37.64±0.15 | 47.33±0.02 | 43.2±0.011 | 41.06±0.01 |
|  | SFW (g) | 6.59±0.01 | 6.1±0.011 | 5.87±0.01 | 5.77±0.01 | 6.42±0.01 | 6.24±0.011 |
|  | RFW (g) | 4.51±0.01 | 3.94±0.006 | 3.7±0.01 | 4.6±0.02 | 4.3±0.01 | 4.11±0.01 |
|  | SDW (g) | 1.61±1.16 | 1.33±0.01 | 1.25±0.01 | 1.7±0.016 | 1.51±0.01 | 1.4±0.01 |
|  | RDW (g) | 1.3±0.016 | 1.14±0.01 | 1.07±0.01 | 1.35±0.01 | 1.18±0.01 | 1.097±0.01 |
|  |  |  |  |  |  |  |  |
| **GEMMIZA 7** | Ph (cm) | 36.4±0.01 | 25.35±0.01 | 22.5±0.12 | 39.7±0.012 | 32.1±0.01 | 29.59±0.013 |
|  | SFW (g) | 6.2±0.01 | 5.46±0.011 | 5.19±0.012 | 6.38±0.01 | 5.93±0.01 | 5.76±0.01 |
|  | RFW (g) | 3.65±0.01 | 3.29±0.01 | 3.18±0.025 | 3.92±0.016 | 3.5±0.015 | 3.37±0.01 |
|  | SDW (g) | 0.88±0.01 | 0.54±0.01 | 0.61±0.01 | 1.07±0.016 | 0.79±0.01 | 0.77±0.026 |
|  | RDW (g) | 0.87±0.016 | 0.66±0.01 | 0.6±0.01 | 0.92±0.01 | 0.71±0.01 | 0.68±0.01 |
|  |  |  |  |  |  |  |  |
| **GEMMIZA 9** | Ph (cm) | 41.6±0.02 | 34.62±0.02 | 31.55±0.1 | 43.1±0.015 | 38.89±0.015 | 36.72±0.025 |
|  | SFW (g) | 6.59±0.01 | 6.1±0.011 | 5.77±0.01 | 6.86±0.01 | 6.4±0.012 | 6.24±0.01 |
|  | RFW (g) | 4.17±0.016 | 3.7±0.016 | 3.54±0.021 | 4.8±0.012 | 3.94±0.01 | 3.71±0.015 |
|  | SDW (g) | 1.42±0.01 | 1.15±0.01 | 1.04±0.01 | 1.55±0.01 | 1.37±0.01 | 1.31±0.01 |
|  | RDW (g) | 1.25±0.01 | 1.1±0.021 | 0.97±0.01 | 1.28±0.01 | 1.2±0.021 | 1.17±0.015 |
|  |  |  |  |  |  |  |  |
| **GEMMIZA 10** | Ph (cm) | 38.42±0.025 | 31.44±0.02 | 25.75±0.021 | 40.3±0.015 | 36.68±0.021 | 34.19±0.01 |
|  | SFW (g) | 5.9±0.01 | 4.92±0.011 | 4.63±0.02 | 6.19±0.015 | 5.36±0.01 | 5.17±0.01 |
|  | RFW (g) | 3.7±0.01 | 3.58±0.015 | 3.44±0.01 | 3.87±0.015 | 3.62±0.01 | 3.52±0.011 |
|  | SDW (g) | 1.1±0.01 | 0.8±0.01 | 0.74±0.011 | 1.2±0.01 | 0.94±0.01 | 0.87±0.01 |
|  | RDW (g) | 1.02±0.01 | 0.82±0.01 | 0.74±0.01 | 1.07±0.01 | 0.95±0.021 | 0.87±0.01 |
|  |  |  |  |  |  |  |  |
| **GEMMIZA 11** | Ph (cm) | 40.47±0.021 | 31.69±0.01 | 26.22±0.015 | 42.28±0.015 | 38.41±0.01 | 33.5±0.011 |
|  | SFW (g) | 5.93±0.01 | 5.44±0.11 | 5.18±0.01 | 6.11±0.012 | 5.71±0.01 | 5.44±0.013 |
|  | RFW (g) | 3.87±0.02 | 3.49±0.015 | 3.35±0.06 | 4.12±0.01 | 3.61±0.02 | 3.55±0.015 |
|  | SDW (g) | 1.22±0.01 | 1.1±0.01 | 1.03±0.01 | 1.34±0.016 | 1.19±0.016 | 1.17±0.006 |
|  | RDW (g) | 1.07±0.016 | 0.87±0.015 | 0.82±0.01 | 1.13±0.01 | 1.03±0.01 | 0.98±0.016 |
|  |  |  |  |  |  |  |  |
| **GEMMIZA 12** | Ph (cm) | 41.05±0.02 | 34.35±0.01 | 29.8±0.011 | 42.69±0.01 | 37.41±0.011 | 34.5±0.02 |
|  | SFW (g) | 6.2±0.01 | 5.56±0.16 | 5.19±0.01 | 6.38±0.011 | 5.94±0.01 | 5.76±0.012 |
|  | RFW (g) | 4.04±0.012 | 3.7±0.016 | 3.4±0.011 | 4.16±0.01 | 3.9±0.01 | 3.66±0.02 |
|  | SDW (g) | 1.38±0.01 | 1.06±0.01 | 0.96±0.01 | 1.43±0.016 | 1.35±0.01 | 1.27±0.01 |
|  | RDW (g) | 1.2±0.01 | 0.97±0.01 | 0.92±0.01 | 1.23±0.016 | 1.17±0.01 | 1.14±0.006 |

**Table S2:** Variation of the examined Changes in photosynthetic pigment and gas exchange parameters in the examined wheat cultivars under different NaCl concentrations alone or with addition of GABA treatments. Means ± SEs, *n* = 4, at *P* < 0.05 according to Duncan’s test.

| **Wheat cultivars** | **Mean ±SD** | **Time (week)** | **Control** | **S1** | **S2** | **GABA** | **GABA +S1** | **GABA+S2** |
| --- | --- | --- | --- | --- | --- | --- | --- | --- |
| **Misr 2** | Leaf chl. (mg/g F W) | 1 Week | 17.92±0.01 | 13.3±0.01 | 11.94±0.006 | 18.1±0.01 | 17.64±0.006 | 17.41±0.01 |
|  |  | 2 Weeks | 17.85±0.006 | 13.23±0.09 | 11.65±0.01 | 18.27±0.02 | 17.2±0.24 | 17.03±0.015 |
|  | Pn (µmol(CO2) m–2 s-1) | 1 Week | 5.66±0.01 | 4.76±0.01 | 4.3±0.01 | 5.5±0.01 | 5.42±0.02 | 5.33±0.02 |
|  |  | 2 Weeks | 6.62±0.015 | 4.56±0.01 | 4.1±0.015 | 5.59±0.021 | 5.21±0.015 | 5.24±0.02 |
|  | Gs (mmol H2O m-2s-2) | 1 Week | 24.32±0.006 | 22.81±0.015 | 22.65±0.01 | 24.49±0.02 | 25.25±1.15 | 23.7±0.01 |
|  |  | 2 Weeks | 24.3±0.026 | 22.53±0.01 | 22.42±0.01 | 24.56±0.015 | 24.65±0.015 | 23.26±0.015 |
|  | Tr ( mmolm−2 s−1) | 1 Week | 13.25±0.01 | 10.31±0.01 | 10.06±0.01 | 13.75±0.015 | 12.9±0.015 | 12.63±0.01 |
|  |  | 2 Weeks | 13.22±0.01 | 10.11±0.01 | 9.99±0.021 | 13.83±0.015 | 12.72±0.01 | 12.44±0.01 |
|  | EL (%) | 1 Week | 23.91±0.01 | 41.2±0.005 | 49.2±0.01 | 23.71±0.01 | 39.82±0.03 | 47.66±0.01 |
|  |  | 2 Weeks | 24.06±0.01 | 41.17±0.01 | 49.06±0.01 | 23.78±0.01 | 33.25±0.01 | 44.67±0.015 |
|  | LWP (MPa) | 1 Week | 0.31±0.006 | 0.72±0.01 | 0.79±0.02 | 0.4±0.01 | 0.83±0.01 | 0.91±0.01 |
|  |  | 2 Weeks | 0.31±0.01 | 0.82±0.01 | 0.87±0.01 | 0.43±0.01 | 0.87±0.006 | 0.95±0.01 |
|  |  |  |  |  |  |  |  |  |
| **SAKHA 94** | Leaf chl. (mg/g F W) | 1 Week | 18.07±0.01 | 14.3±0.01 | 12.65±0.01 | 18.3±0.01 | 17.95±0.01 | 17.55±0.01 |
|  |  | 2 Weeks | 18.05±0.015 | 14.1±0.01 | 12.43±0.01 | 18.38±0.01 | 17.54±0.01 | 17.35±0.006 |
|  | Pn (µmol(CO2) m–2 s-1) | 1 Week | 6.92±0.01 | 5.92±0.02 | 5.75±0.015 | 6.69±0.015 | 6.44±0.01 | 6.05±0.015 |
|  |  | 2 Weeks | 6.92±0.009 | 5.57±0.01 | 5.34±0.01 | 6.78±0.006 | 6.24±0.01 | 5.88±0.01 |
|  | Gs (mmol H2O m-2s-2) | 1 Week | 26.71±0.0015 | 23.25±0.01 | 19.76±0.01 | 28.81±0.01 | 26.53±25.41 | 25.48±0.015 |
|  |  | 2 Weeks | 26.7±0.01 | 23.04±0.015 | 19.57±0.02 | 28.95±0.01 | 26.41±0.01 | 25.24±0.01 |
|  | Tr ( mmolm−2 s−1) | 1 Week | 21..05±0.011 | 12.54±0.01 | 15.82±0.015 | 21.31±0.011 | 20.78±0.015 | 20.52±0.01 |
|  |  | 2 Weeks | 21.08±0.104 | 12.33±0.01 | 15.58±0.01 | 21.48±0.01 | 20.54±0.01 | 20.33±0.01 |
|  | EL (%) | 1 Week | 26.3±0.01 | 49.9±0.01 | 53.37±0.16 | 26.3±0.015 | 58.4±0.01 | 64.1±0.01 |
|  |  | 2 Weeks | 26.45±0.015 | 58.26±0.02 | 64.01±0.015 | 25.81±0.01 | 49.9±0.01 | 53.28±0.01 |
|  | LWP (MPa) | 1 Week | 0.64±0.006 | 0.82±0.01 | 0.95±0.01 | 0.75±0.01 | 1.17±0.01 | 1.35±0.01 |
|  |  | 2 Weeks | 0.62±0.01 | 0.85±0.01 | 0.99±0.01 | 0.8±0.01 | 1.23±0.01 | 1.45±0.01 |
|  |  |  |  |  |  |  |  |  |
| **SAKHA 95** | Leaf chl. (mg/g F W) | 1 Week | 18.15±0.01 | 15.45±0.01 | 13.65±0.01 | 18.27±0.01 | 17.8±0.01 | 17.54±0.01 |
|  |  | 2 Weeks | 18.11±0.017 | 15.33±0.01 | 13.42±0.015 | 18.33±0.01 | 17.65±0.015 | 17.21±0.015 |
|  | Pn (µmol(CO2) m–2 s-1) | 1 Week | 6.6±0.015 | 5.72±0.015 | 5.44±0.03 | 6.82±0.015 | 6.3±0.011 | 6.13±0.01 |
|  |  | 2 Weeks | 6.63±0.021 | 5.4±0.01 | 5.19±0.015 | 6.89±0.02 | 6.02±0.015 | 5.97±0.011 |
|  | Gs (mmol H2O m-2s-2) | 1 Week | 25.41±0.006 | 18.94±0.01 | 16.24±0.01 | 27.34±0.01 | 23.82±0.015 | 20.7±0.006 |
|  |  | 2 Weeks | 25.41±0.032 | 18.64±0.01 | 16.04±0.02 | 27.43±0.01 | 23.55±0.02 | 20.54±0.01 |
|  | Tr ( mmolm−2 s−1) | 1 Week | 20.8±0.01 | 16.45±0.015 | 16.17±0.015 | 20.89±0.015 | 20.12±0.015 | 19.87±0.01 |
|  |  | 2 Weeks | 20.76±0.044 | 16.25±0.01 | 16.02±0.012 | 20.98±0.01 | 19.9±0.01 | 19.62±0.02 |
|  | EL (%) | 1 Week | 26.1±0.01 | 53.4±0.01 | 67.12±0.01 | 25.7±0.015 | 50.3±0.015 | 61.9±0.01 |
|  |  | 2 Weeks | 26.21±0.01 | 55.38±0.015 | 63.02±0.015 | 25.69±0.015 | 50.2±0.01 | 61.74±0.047 |
|  | LWP (MPa) | 1 Week | 0.55±0.01 | 0.88±0.01 | 0.94±0.015 | 0.64±0.02 | 1.25±0.015 | 1.43±0.010.01 |
|  |  | 2 Weeks | 0.54±0.01 | 0.95±0.01 | 1.03±0.01 | 0.71±0.01 | 1.34±0.010.01 | 1.54±0.010.015 |
|  |  |  |  |  |  |  |  |  |
| **GEMMIZA 7** | Leaf chl. (mg/g F W) | 1 Week | 23.86±0.006 | 44.2±0.01 | 52.6±0.01 | 21.6±0.01 | 41.14±0.01 | 50.26±0.01 |
|  |  | 2 Weeks | 23.94±0.01 | 46.11±0.01 | 54.29±0.56 | 17.31±0.01 | 43.01±0.011 | 51.2±0.01 |
|  | Pn (µmol(CO2) m–2 s-1) | 1 Week | 23.21±0.01 | 21.42±0.01 | 20.96±0.01 | 23.42±0.026 | 22.95±0.01 | 22.54±0.015 |
|  |  | 2 Weeks | 23.23±0.025 | 21.2±0.01 | 20.56±0.01 | 23.57±0.015 | 22.65±0.012 | 22.35±0.01 |
|  | Gs (mmol H2O m-2s-2) | 1 Week | 23.21±0.01 | 21.42±0.01 | 20.96±0.01 | 23.42±0.026 | 22.95±0.01 | 22.54±0.015 |
|  |  | 2 Weeks | 23.23±0.25 | 21.21±0.01 | 20.56±0.01 | 23.57±0.015 | 22.64±0.011 | 22.35±0.01 |
|  | Tr ( mmolm−2 s−1) | 1 Week | 16.23±0.01 | 14.9±0.01 | 13.52±0.015 | 16.42±0.015 | 15.36±0.01 | 15.2±0.01 |
|  |  | 2 Weeks | 16.23±0.01 | 14.97±0.025 | 13.34±0.01 | 16.5±0.01 | 15.15±0.11 | 15.03±0.01 |
|  | EL (%) | 1 Week | 23.6±0.01 | 43.15±0.01 | 51.26±0.01 | 23.86±0.015 | 44.2±0.01 | 52.62±0.02 |
|  |  | 2 Weeks | 23.6±0.01 | 43.01±0.006 | 51.2±0.01 | 23.94±0.006 | 44.11±0.015 | 52.17±0.58 |
|  | LWP (MPa) | 1 Week | 0.42±0.01 | 0.76±0.01 | 0.92±0.01 | 0.5±0.01 | 0.93±0.01 | 0.98±0.01 |
|  |  | 2 Weeks | 0.41±0.01 | 0.83±0.01 | 0.98±0.01 | 0.62±0.01 | 1.01±0.006 | 1.07±0.01 |
|  |  |  |  |  |  |  |  |  |
| **GEMMIZA 9** | Leaf chl. (mg/g F W) | 1 Week | 19.71±0.01 | 17.14±0.12 | 16.87±0.015 | 19.7±0.01 | 18.76±0.01 | 18.52±0.01 |
|  |  | 2 Weeks | 19.74±0.01 | 16.99±0.015 | 16.65±0.01 | 19.82±0.01 | 18.44±0.025 | 18.32±0.025 |
|  | Pn (µmol(CO2) m–2 s-1) | 1 Week | 25.03±0.01 | 24.64±0.01 | 22.9±0.006 | 25.4±0.015 | 24.73±0.01 | 24.52±0.015 |
|  |  | 2 Weeks | 24.98±0.015 | 23.25±0.01 | 22.43±0.02 | 25.5±0.015 | 24.42±0.01 | 24.32±0.026 |
|  | Gs (mmol H2O m-2s-2) | 1 Week | 25.03±0.01 | 23.64±0.01 | 22.8±0.006 | 25.41±0.015 | 24.73±0.01 | 24.52±0.015 |
|  |  | 2 Weeks | 24.98±0.015 | 23.25±0.01 | 22.43±0.02 | 25.5±0.015 | 24.42±0.01 | 24.32±0.026 |
|  | Tr ( mmolm−2 s−1) | 1 Week | 19.58±0.015 | 16.24±0.01 | 15.74±0.01 | 19.81±0.01 | 19.32±0.015 | 18.89±0.02 |
|  |  | 2 Weeks | 19.57±0.025 | 16.06±0.02 | 15.65±0.98 | 19.89±0.025 | 19.14±0.015 | 18.72±0.02 |
|  | EL (%) | 1 Week | 26.9±0.01 | 51.3±0.01 | 55.14±0.015 | 23.41±0.038 | 48.27±0.02 | 51.51±0.015 |
|  |  | 2 Weeks | 27.03±0.006 | 54.26±0.01 | 59.85±0.01 | 23.35±0.01 | 48.25±0.01 | 51.53±0.01 |
|  | LWP (MPa) | 1 Week | 0.59±0.01 | 0.94±0.01 | 1.02±0.01 | 0.65±0.01 | 1.12±0.01 | 1.26±0.01 |
|  |  | 2 Weeks | 0.61±0.01 | 1.03±0.01 | 1.91±0.01 | 0.77±0.01 | 1.19±0.006 | 1.23±0.02 |
|  |  |  |  |  |  |  |  |  |
| **GEMMIZA 10** | Leaf chl. (mg/g F W) | 1 Week | 17.27±0.01 | 16.67±0.15 | 14.66±0.01 | 17.42±0.012 | 16.81±0.01 | 16.73±0.011 |
|  |  | 2 Weeks | 17.24±0.01 | 16.45±0.015 | 14.42±0.006 | 17.55±0.016 | 16.54±0.01 | 16.45±0.015 |
|  | Pn (µmol(CO2) m–2 s-1) | 1 Week | 5.95±0.01 | 4.82±0.006 | 4.66±0.01 | 6.02±0.01 | 5.72±0.015 | 5.58±0.01 |
|  |  | 2 Weeks | 5.96±0.025 | 4.56±0.21 | 4.33±0.021 | 6.12±0.01 | 5.36±0.01 | 5.21±1.65 |
|  | Gs (mmol H2O m-2s-2)  Tr ( mmolm−2 s−1) | 1 Week | 25.08±0.01 | 24.51±0.01 | 24.06±0.016 | 25.21±0.016 | 24.91±0.01 | 24.72±0.015 |
|  |  | 2 Weeks | 24.1±0.02 | 24.33±0.01 | 22.96±0.01 | 25.27±0.015 | 24.65±0.015 | 24.42±0.01 |
|  |  | 1 Week | 20.24±0.01 | 16.92±0.16 | 17.42±0.99 | 20.39±0.015 | 18.8±0.01 | 18.95±0.015 |
|  | EL (%) | 2 Weeks | 20.23±0.015 | 18.03±0.01 | 17.54±0.01 | 20.53±0.015 | 18.62±0.011 | 18.76±0.01 |
|  |  | 1 Week | 24.71±±0.01 | 45.17±0.015 | 50.7±0.015 | 22.6±0.015 | 43.17±0.01 | 47.8±0.01 |
|  |  | 2 Weeks | 24.84±0.01 | 49.7±0.01 | 53.55±0.016 | 23.73±0.01 | 45.01±0.01 | 50.55±0.01 |
|  | LWP (MPa) | 1 Week | 0.43±0.01 | 1.04±0.01 | 1.13±0.01 | 0.49±0.01 | 1.21±0.015 | 1.36±0.01 |
|  |  | 2 Weeks | 0.42±0.01 | 1.11±0.01 | 1.21±0.045 | 0.55±0.01 | ±1.35±0.045 | 1.47±0.025 |
|  |  |  |  |  |  |  |  |  |
| **GEMMIZA 11** | Leaf chl. (mg/g F W) | 1 Week | 17.55±0.015 | 14.3±0.01 | 12.87±0.006 | 17.86±0.01 | 17.4±0.015 | 17.29±0.01 |
|  |  | 2 Weeks | 17.56±0.01 | 14.07±0.01 | 12.64±0.01 | 17.63±0.57 | 17.32±0.08 | 17.1±0.015 |
|  | Pn (µmol(CO2) m–2 s-1) | 1 Week | 5.84±0.015 | 4.81±0.01 | 4.76±0.01 | 5.91±0.01 | 5.65±0.01 | 5.34±0.015 |
|  |  | 2 Weeks | 5.82±0.01 | 4.39±0.006 | 4.43±0.02 | 5.93±0.026 | 5.41±0.099 | 5.2±0.01 |
|  | Gs (mmol H2O m-2s-2) | 1 Week | 21.35±0.01 | 20.18±0.01 | 20.01±0.12 | 21.41±0.01 | 20.87±0.015 | 20.47±0.015 |
|  |  | 2 Weeks | 21.29±0.02 | 20.03±0.015 | 19.88±0.01 | 20.63±0.01 | 20.63±0.01 | 20.27±0.01 |
|  | Tr ( mmolm−2 s−1) | 1 Week | 19.25±0.01 | 14.51±0.015 | 13.74±0.15 | 19.42±0.015 | 18.8±0.01 | 18.20.11 |
|  |  | 2 Weeks | 19.23±0.015 | 14.24±0.01 | 13.62±0.2 | 19.66±1.015 | 18.7±0.015 | 18.14±0.006 |
|  | EL (%) | 1 Week | 25.12±0.015 | 46.5±0.015 | 52.25±0.03 | 24.65±0.01 | 42.47±0.07 | 49.21±0.01 |
|  |  | 2 Weeks | 25.22±0.01 | 50.51±0.06 | 54.12±0.01 | 24.86±0.015 | 46.4±0.01 | 52.19±0.006 |
|  | LWP (MPa) | 1 Week | 0.58±0.015 | 1.05±0.01 | 1.09±0.01 | 0.64±0.015 | 1.43±0.01 | 1.35±0.015 |
|  |  | 2 Weeks | 0.55±0.015 | 1.15±0.011 | 1.18±0.01 | 0.75±0.015 | 1.58±0.02 | 1.75±0.01 |
|  |  |  |  |  |  |  |  |  |
| **GEMMIZA 12** | Leaf chl. (mg/g F W) | 1 Week | 20.15±0.01 | 18.9±0.015 | 17.6±0.015 | 21.63±0.01 | 20.03±0.006 | 19.87±0.01 |
|  |  | 2 Weeks | 20.12±0.02 | 18.42±0.01 | 17.24±0.196 | 21.71±0.01 | 19.77±0.006 | 19.64±0.01 |
|  | Pn (µmol(CO2) m–2 s-1) | 1 Week | 6.29±0.015 | 5.61±0.01 | 5.5±0.01 | 6.5±0.01 | 6.13±0.01 | 5.94±0.01 |
|  |  | 2 Weeks | 6.27±0.01. | 5.34±0.02 | 5.2±0.01 | 6.58±0.02 | 5.87±0.015 | 5.42±0.01 |
|  | Gs (mmol H2O m-2s-2) | 1 Week | 26.32±0.02 | 23.8±0.07 | 22.92±0.01 | 26.43±0.02 | 25.95±0.01 | 25.65±0.026 |
|  |  | 2 Weeks | 26.33±0.015 | 23.52±0.02 | 22.67±0.01 | 26.54±0.015 | 25.54±0.015 | 25.36±0.08 |
|  | Tr ( mmolm−2 s−1) | 1 Week | 18.79±0.025 | 15.4±0.01 | 14.29±0.01 | 19.6±0.016 | 17.94±0.015 | 17.12±0.01 |
|  |  | 2 Weeks | 18.76±0.015 | 15.23±0.01 | 14.11±0.01 | 19.85±0.01 | 18.01±0.015 | 17.01±0.006 |
|  | EL (%) | 1 Week | 25.85±0.01 | 48.22±0.01 | 54.13±0.015 | 23.65±0.01 | 45.67±0.015 | 50.11±0.016 |
|  |  | 2 Weeks | 25.98±0.01 | 53.53±0.01 | 57.06±0.01 | 25.66±0.01 | 47.52±0.006 | 54.37±0.006 |
|  | LWP (MPa) | 1 Week | 0.58±0.015 | 1.09±0.01 | 1.25±0.01 | 0.62±0.01 | 1.39±0.01 | 1.56±0.01 |
|  |  | 2 Weeks | 0.56±0.01 | 1.14±0.025 | 1.38±0.015 | 0.7±0.01 | 1.46±0.01 | 1.68±0.015 |

***Table S3: Variation of the examined the stress-induced biomarkers in different wheat cultivars under different NaCl concentrations alone or with addition of GABA treatments. Means ± SEs, n = 4, at P < 0.05 according to Duncan’s test.***

| **Wheat cultivars** | **Mean ±SD** | **Time (week)** | **Control** | **S1** | **S2** | **GABA** | **GABA +S1** | **GABA+S2** |
| --- | --- | --- | --- | --- | --- | --- | --- | --- |
| **Misr 2** | MDA (µmol/g F W) | 1 Week | 2.38±0.006 | 3.9±0.01 | 4.21±0.015 | 2.29±0.01 | 3.51±0.01 | 3.92±0.015 |
|  |  | 2 Weeks | 2.37±0.01 | 4.02±0.01 | 4.38±0.01 | 2.07±0.01 | 3.62±0.01 | 4.02±0.01 |
|  | H2O2 (µmol/g F W) | 1 Week | 1.52±0.01 | 2.06±0.015 | 2.35±0.012 | 1.46±0.01 | 1.87±0.01 | 2.01±0.01 |
|  |  | 2 Weeks | 1.54±0.01 | 2.15±0.01 | 2.40±0.006 | 1.29±0.01 | 1.81±0.01 | 1.99±0.01 |
|  | Proline (µmol g-1 FW) | 1 Week | 5.42±0.01 | 9.04±0.01 | 9.17±0.01 | 5.7±0.01 | 9.25±0.011 | 9.93±0.01 |
|  |  | 2 Weeks | 5.42±0.01 | 1.24±0.006 | 1.42±0.01 | 5.97±0.01 | 9.44±0.01 | 10.13±0.01 |
|  | SOD (Ug-1FW) | 1 Week | 26.8±0.01 | 36.92±0.006 | 42.56±0.01 | 27.35±0.01 | 41.61±0.01 | 46.72±0.01 |
|  |  | 2 Weeks | 26.81±0.01 | 28.1±0.02 | 37.43±0.015 | 27.62±0.02 | 45.57±0.01 | 50.3±0.006 |
|  | CAT (Ug-1FW) | 1 Week | 19.95±0.01 | 22.74±0.01 | 24.66±0.01 | 15.2±0.01 | 25.82±0.01 | 29.85±0.049 |
|  |  | 2 Weeks | 19.97±0.015 | 23.62±0.02 | 25.62±0.006 | 15.17±0.16 | 26.22±0.026 | 30.14±0.015 |
|  | APX (Ug-1FW) | 1 Week | 19.7±0.01 | 42.87±0.58 | 47.41±0.015 | 19.7±0.015 | 49.81±0.01 | 53.61±0.011 |
|  |  | 2 Weeks | 19.65±0.01 | 47.43±0.015 | 49.51±0.01 | 19.54±0.015 | 50.6±0.01 | 55.28±0.011 |
|  |  |  |  |  |  |  |  |  |
| **SAKHA 94** | MDA (µmol/g F W) | 1 Week | 3.16±0.006 | 5.77±0.01 | 8.65±0.01 | 2.85±0.01 | 4.53±0.015 | 7.9±0.015 |
|  |  | 2 Weeks | 3.21±0.01 | 6.28±0.02 | 9.98±0.015 | 2.94±0.01 | 5.94±0.01 | 9.12±0.21 |
|  | H2O2 (µmol/g F W) | 1 Week | 2.14±0.01 | 4.15±0.01 | 4.59±0.015 | 1.9±0.01 | 3.97±0.015 | 4.19±0.026 |
|  |  | 2 Weeks | 2.21±0.015 | 4.05±0.01 | 4.32±0.01 | 1.81±0.025 | 3.85±0.01 | 4.29±0.006 |
|  | Proline (µmol g-1 FW) | 1 Week | 6.83±0.015 | 11.05±0.012 | 11.97±0.01 | 6.6±0.58 | 11.51±0.01 | 12.6±0.01 |
|  |  | 2 Weeks | 6.75±0.01 | 11.44±0.01 | 12.13±0.11 | 7.04±0.01 | 11.67±0.01 | 12.84±0.01 |
|  | SOD (Ug-1FW) | 1 Week | 31.45±0.01 | 44.31±0.01 | 47.5±0.006 | 31.5±0.01 | 55.7±0.01 | 60.34±0.015 |
|  |  | 2 Weeks | 31.43±0.015 | 42.45±0.006 | 43.58±0.025 | 30.54±0.02 | 53.21±0.015 | 56.41±0.01 |
|  | CAT (Ug-1FW) | 1 Week | 31.06±0.01 | 43.75±0.011 | 52.19±0.006 | 30.76±0.01 | 53.14±0.01 | 54.15±0.006 |
|  |  | 2 Weeks | 31.04±0.02 | 44.04±0.026 | 53.13±0.012 | 30.41±0.01 | 54.12±0.026 | 55.6±0.02 |
|  | APX (Ug-1FW) | 1 Week | 33.64±0.01 | 117.8±0.02 | 123.6±0.02 | 33.8±0.01 | 125.31±0.015 | 142.55±0.01 |
|  |  | 2 Weeks | 33.62±0.02 | 118.5±0.02 | 129.49±0.006 | 33.72±0.025 | 128.62±0.02 | 150.31±0.02 |
|  |  |  |  |  |  |  |  |  |
| **SAKHA 95** | MDA (µmol/g F W) | 1 Week | 3.05±0.01 | 8.65±0.006 | 8.94±0.01 | 2.83±0.015 | 7.22±0.01 | 7.66±0.01 |
|  |  | 2 Weeks | 3.14±0.015 | 8.82±0.01 | 9.1±0.15 | 2.88±0.01 | 7.39±0.01 | 7.71±0.01 |
|  | H2O2 (µmol/g F W) | 1 Week | 2.09±0.01 | 3.95±0.01 | 4.11±0.01 | 1.84±0.01 | 3.61±0.01 | 3.84±0.015 |
|  |  | 2 Weeks | 2.18±0.01 | 4.08±0.006 | 4.23±0.025 | 2.04±0.01 | 3.74±0.015 | 3.93±0.061 |
|  | Proline (µmol g-1 FW) | 1 Week | 6.96±0.01 | 11.05±0.01 | 12.05±0.006 | 7.12±0.01 | 11.96±0.01 | 12.45±0.01 |
|  |  | 2 Weeks | 7.02±0.01 | 11.36±0.011 | 12.13±0.016 | 7.22±0.01 | 12.1±0.01 | 12.55±0.02 |
|  | SOD (Ug-1FW) | 1 Week | 30.65±0.01 | 53.15±0.01 | 59.7±0.01 | 30.8±0.015 | 58.96±0.006 | 62.4±0.01 |
|  |  | 2 Weeks | 30.62±0.015 | 51.62±0.01 | 54.67±0.01 | 29.54±0.16 | 55.25±0.015 | 60.4±0.02 |
|  | CAT (Ug-1FW) | 1 Week | 21.43±0.006 | 33.61±0.01 | 36.79±0.01 | 21.69±0.015 | 37.61±0.01 | 27.49±0.01 |
|  |  | 2 Weeks | 21.42±0.015 | 35.6±0.015 | 37.12±0.011 | 21.52±0.015 | 37.64±0.12 | 28.96±0.01 |
|  | APX (Ug-1FW) | 1 Week | 23.14±0.015 | 114.78±0.015 | 125.5±0.015 | 36.25±0.006 | 120.7±0.01 | 132.7±0.03 |
|  |  | 2 Weeks | 36.14±0.01 | 119.4±0.025 | 136.2±0.015 | 36.34±0.017 | 122.6±0.6 | 142.95±0.6 |
|  |  |  |  |  |  |  |  |  |
| **GEMMIZA 7** | MDA (µmol/g F W) | 1 Week | 2.13±0.01 | 4.76±0.015 | 5.17±0.01 | 1.88±0.01 | 4.45±0.006 | 4.81±0.01 |
|  |  | 2 Week | 2.31±0.01 | 4.84±0.015 | 5.74±0.01 | 1.72±0.01 | 4.55±0.006 | 4.98±0.01 |
|  | H2O2 (µmol/g F W) | 1 Week | 1.54±0.01 | 2.18±0.01 | 2.43±0.006 | 1.38±0.01 | 1.67±0.021 | 1.94±0.01 |
|  |  | 2 Weeks | 1.67±0.01 | 2.27±0.11 | 2.53±0.01 | 1.37±0.01 | 1.96±0.01 | 2.14±0.01 |
|  | Proline (µmol g-1 FW) | 1 Week | 5.52±0.01 | 9.24±0.01 | 9.86±0.01 | 5.7±0.015 | 9.62±0.01 | 10.11±0.015 |
|  |  | 2 Weeks | 5.66±0.01 | 3.34±0.01 | 9.97±0.01 | 5.85±0.01 | 9.84±0.011 | 10.35±0.01 |
|  | SOD (Ug-1FW) | 1 Week | 57.72±0.011 | 37.25±0.01 | 44.7±0.01 | 26.2±0.01 | 40.6±0.01 | 51.6±0.01 |
|  |  | 2 Weeks | 25.74±0.01 | 33.6±0.02 | 42.87±1.013 | 25.94±0.01 | 38.6±0.02 | 50.13±0.011 |
|  | CAT (Ug-1FW) | 1 Week | 17.74±0.01 | 23.81±0.01 | 26.59±0.01 | 19.41±0.01 | 24.38±0.015 | 27.6±0.01 |
|  |  | 2 Weeks | 19.72±0.02 | 24.81±0.015 | 27.35±0.01 | 19.24±0.025 | 25.61±0.015 | 29.38±0.015 |
|  | APX (Ug-1FW) | 1 Week | 24.17±0.026 | 54.3±0.011 | 57.9±0.01 | 24.3±0.01 | 68.23±0.025 | 78.6±0.01 |
|  |  | 2 Weeks | 24.12±0.020 | 46.49±0.01 | 60.5±0.015 | 24.25±0.015 | 71.54±0.02 | 84.26±0.025 |
|  |  |  |  |  |  |  |  |  |
| **GEMMIZA 9** | MDA (µmol/g F W) | 1 Week | 2.75±0.01 | 5.27±0.01 | 6.08±0.015 | 2.29±0.01 | 4.92±0.006 | 5.66±0.015 |
|  |  | 2 Weeks | 2.9±0.01 | 5.38±0.14 | 8.91±0.015 | 2.16±0.02 | 4.96±0.015 | 8.11±0.015 |
|  | H2O2 (µmol/g F W) | 1 Week | 1.98±0.01 | 2.15±0.01 | 2.29±0.035 | 1.7±0.015 | 2.02±0.01 | 2.16±0.021 |
|  |  | 2 Weeks | 2.05±0.01 | 2.19±0.011 | 2.41±0.053 | 1.94±0.01 | 2.13±0.012 | 2.28±0.05 |
|  | Proline (µmol g-1 FW) | 1 Week | 6.43±0.01 | 10.24±0.01 | 11.13±0.01 | 6.78±0.01 | 10.52±0.01 | 11.32±0.01 |
|  |  | 2 Weeks | 6.55±0.02 | 10.44±0.01 | 11.25±0.01 | 6.84±0.012 | 10.7±0.01 | 11.45±0.01 |
|  | SOD (Ug-1FW) | 1 Week | 30.89±0.01 | 58.12±0.006 | 60.3±0.01 | 31.05±0.01 | 63.17±0.015 | 68.16±0.021 |
|  |  | 2 Weeks | 30.91±0.01 | 55.12±0.025 | 56.2±0.025 | 28.7±0.015 | 60.58±0.015 | 64.14±0.01 |
|  | CAT (Ug-1FW) | 1 Week | 17.93±0.01 | 29.7±0.01 | 30.94±0.01 | 17.8±0.01 | 29.62±0.01 | 31.2±0.01 |
|  |  | 2 Weeks | 17.9±0.02 | 30.12±0.02 | 32.54± 0.02 | 17.09±0.01 | 30.5±0.01 | 34.5±0.01 |
|  | APX (Ug-1FW) | 1 Week | 31.18±0.01 | 88.73±0.015 | 92.44±0.015 | 31.43±0.02 | 96.69±0.01 | 109.27±0.01 |
|  |  | 2 Weeks | 31.22±0.06 | 90.25±0.01 | 94.7±0.01 | 31.3±0.01 | 102.5±0.015 | 112.37±0.015 |
|  |  |  |  |  |  |  |  |  |
| **GEMMIZA 10** | MDA (µmol/g F W) | 1 Week | 2.42±0.01 | 5.82±0.53 | 6.31±0.01 | 1.99±0.01 | 5.43±0.006 | 6.17±0.01 |
|  |  | 2 Weeks | 2.53±0.02 | 6.01±0.01 | 6.43±0.01 | 2.76±0.01 | 5.56±0.26 | 6.28±0.014 |
|  | H2O2 (µmol/g F W) | 1 Week | 1.68±0.015 | 2.31±0.006 | 2.64±0.015 | 1.39±0.01 | 2.12±0.01 | 2.37±0.01 |
|  |  | 2 Weeks | 1.76±0.01 | 2.47±0.01 | 2.74±0.01 | 1.26±0.01 | 2.26±0.01 | 2.47±0.01 |
|  | Proline (µmol g-1 FW)  SOD (Ug-1FW) | 1 Week | 6.33±0.015 | 9.36±0.01 | 9.58±0.015 | 6.48±0.015 | 9.6±0.015 | 9.76±0.31 |
|  |  | 2 Weeks | 6.43±0.01 | 9.49±0.01 | 9.65±0.038 | 6.6±0.01 | 9.74±0.01 | 10.09±0.01 |
|  |  | 1 Week | 28.65±0.01 | 39.51±0.01 | 40.15±0.015 | 30.31±0.01 | 42.2±0.01 | 47.6±0.015 |
|  | CAT (Ug-1FW) | 2 Weeks | 28.65±0.01 | 36.27±0.58 | 37019±0.01 | 29.44±0.01 | 39.84±0.01 | 44.55±0.02 |
|  |  | 1 Week | 17.17±0.01 | 27.7±0.015 | 28.15±0.006 | 17.3±0.01 | 29.6±0.01 | 30.82±0.02 |
|  |  | 2 Weeks | 17.15± 0.015 | 27.96±0.02 | 27.81±0.01 | 17.24±0.011 | 30.43±0.015 | 32.94±0.006 |
|  | APX (Ug-1FW) | 1 Week | 26.77±0.01 | 74.2±0.015 | 81.66±0.026 | 27.41±0.01 | 82.55±0.01 | 93.71±0.015 |
|  |  | 2 Weeks | 26.79±0.015 | 76.4±0.01 | 85.62±0.01 | 27.37±0.025 | 84.25±0.01 | 101.2±0.25 |
|  |  |  |  |  |  |  |  |  |
| **GEMMIZA 11** | MDA (µmol/g F W) | 1 Week | 2.62±0.015 | 6.35±0.015 | 7.92±0.01 | 2.22±0.006 | 6.97±0.01 | 7.01±0.01 |
|  |  | 2 Weeks | 2.77±0.01 | 7.47±0.01 | 8.04±0.01 | 2.17±0.01 | 7.08±0.067 | 7.24±0.01 |
|  | H2O2 (µmol/g F W) | 1 Week | 1.55±0.27 | 2.32±0.006 | 2.71±0.01 | 1.35±0.01 | 2.19±0.01 | 2.46±0.067 |
|  |  | 2 Weeks | 1.68±0.17 | 2.44±0.01 | 2.87±0.015 | 1.44±0.01 | 2.15±0.01 | 2.57±0.01 |
|  | Proline (µmol g-1 FW) | 1 Week | 6.15±0.015 | 9.84±0.01 | 10.23±0.01 | 6.34±0.01 | 10.12±0.01 | 10.75±0.015 |
|  |  | 2 Weeks | 6.29±0.01 | 9.96±0.01 | 10.29±0.01 | 6.44±0.01 | 10.29±0.01 | 10.85±0.01 |
|  | SOD (Ug-1FW) | 1 Week | 28.37±0.01 | 50.4±0.01 | 50.74±0.015 | 28.6±0.006 | 52.3±0.011 | 52.83±0.01 |
|  |  | 2 Weeks | 28.35±0.01 | 48.64±0.011 | 48.61±0.01 | 26.9±0.01 | 50.41±0.01 | 50.7±0.01 |
|  | CAT (Ug-1FW) | 1 Week | 16.52±0.01 | 30.41±0.011 | 34.12±0.01 | 16.72±0.006 | 32.16±0.01 | 34.71±0.01 |
|  |  | 2 Weeks | 16.51±0.006 | 31.03±0.01 | 35.12±0.038 | 16.52±0.02 | 34.25±0.006 | 36.14±0.015 |
|  | APX (Ug-1FW) | 1 Week | 22.61±0.015 | 59.81±0.015 | 62.4±0.01 | 27.14±0.01 | 87.5±0.01 | 52.81±0.015 |
|  |  | 2 Weeks | 22.6±0.015 | 62.91±0.01 | 68.4±0.037 | 27.03±0.026 | 88.4±0.01 | 62.91±0.01 |
|  |  |  |  |  |  |  |  |  |
| **GEMMIZA 12** | MDA (µmol/g F W) | 1 Week | 2.94±0.049 | 7.85±0.015 | 8.23±0.006 | 2.39±0.01 | 7.38±0.01 | 8.95±0.01 |
|  |  | 2 Weeks | 2.98±0.01 | 7.95±0.01 | 8.34±0.015 | 3.41±0.015 | 7.19±0.021 | 7.79±0.01 |
|  | H2O2 (µmol/g F W) | 1 Week | 1.75±0.01 | 2.77±0.01 | 2.99±0.006 | 1.58±0.015 | 2.35±0.015 | 2.78±0.01 |
|  |  | 2 Weeks | 1.74±0.32 | 2.85±0.01 | 3.05±0.02 | 1.43±0.012 | 2.49±0.006 | 2.86±0.01 |
|  | Proline (µmol g-1 FW) | 1 Week | 6.37±0.015 | 10.15±0.01 | 10.32±0.015 | 6.65±0.006 | 10.3±0.01 | 10.97±0.01 |
|  |  | 2 Weeks | 6.5±0.01 | 10.28±0.015 | 10.43±0.015 | 6.76±0.02 | 10.44±0.02 | 11.13±0.01 |
|  | SOD (Ug-1FW) | 1 Week | 29.34±0.011 | 28.61±0.01 | 62.21±0.01 | 29.5±0.01 | 61.25±0.05 | 68.8±0.58 |
|  |  | 2 Weeks | 29.34±0.01 | 25.6±0.01 | 30.29±0.01 | 27.84±0.012 | 60.29±0.015 | 65.87±0.026 |
|  | CAT (Ug-1FW) | 1 Week | 15.63±0.43 | 25.14±0.006 | 28.7±0.006 | 15.84±0.02 | 28.24±.16 | 30.21±0.015 |
|  |  | 2 Weeks | 15.7±0.02 | 26.52±0.015 | 28.72±0.015 | 15.75±0.011 | 29.63±0.026 | 32.58±0.01 |
|  | APX (Ug-1FW) | 1 Week | 29.4±0.01 | 58.67±0.015 | 93.81±0.01 | 29.71±0.02 | 109.76±0.01 | 115.31±0.01 |
|  |  | 2 Weeks | 29.4±0.006 | 60.77±0.01 | 102.6±0.01 | 29.52±0.01 | 115.61±0.01 | 122.83±0.02 |

**Table S4:** Variation of the examined the gene expression levels of salinity tolerant genes in different wheat cultivars under different NaCl concentrations alone or with addition of GABA treatments.

| **MISR 2** | **GeneID** | **Control** | **S1** | **S2** | **GABA** | **GABA +S1** | **GABA+S2** |
| --- | --- | --- | --- | --- | --- | --- | --- |
|  | **NHX1** | 1 | 16.74 | 23.64 | 0.85 | 16.42 | 22.59 |
|  | **DHN3** | 1 | 17.69 | 27.64 | 0.68 | 17.39 | 25.95 |
|  | **GR** | 1 | 18.9 | 26.89 | 0.94 | 18.42 | 24.98 |
|  | **TaSOS1** | 1 | 21.95 | 33.61 | 0.65 | 20.86 | 31.86 |
|  |  |  |  |  |  |  |  |
|  |  |  |  |  |  |  |  |
| **SAKHA 94** | **GeneID** | **Control** | **S1** | **S2** | **GABA** | **GABA +S1** | **GABA+S2** |
|  | **NHX1** | 1 | 22.64 | 34.52 | 0.68 | 22.05 | 33.42 |
|  | **DHN3** | 1 | 21.59 | 26.69 | 0.84 | 20.94 | 25.97 |
|  | **GR** | 1 | 24.55 | 32.41 | 0.59 | 23.46 | 31.75 |
|  | **TaSOS1** | 1 | 12.94 | 14.29 | 0.64 | 12.77 | 13.86 |
|  |  |  |  |  |  |  |  |
|  |  |  |  |  |  |  |  |
| **SAKHA 95** | **GeneID** | **Control** | **S1** | **S2** | **GABA** | **GABA +S1** | **GABA+S2** |
|  | **NHX1** | 1 | 21.64 | 35.62 | 0.64 | 20.64 | 34.89 |
|  | **DHN3** | 1 | 20.41 | 25.67 | 0.98 | 19.42 | 25.31 |
|  | **GR** | 1 | 25.72 | 33.09 | 0.54 | 23.67 | 32.73 |
|  | **TaSOS1** | 1 | 13.08 | 14.62 | 0.56 | 12.43 | 14.12 |
|  |  |  |  |  |  |  |  |
|  |  |  |  |  |  |  |  |
| **GEMMIZA 7** | **GeneID** | **Control** | **S1** | **S2** | **GABA** | **GABA +S1** | **GABA+S2** |
|  | **NHX1** | 1 | 18.1 | 28.34 | 0.98 | 17.56 | 27.62 |
|  | **DHN3** | 1 | 24.69 | 29.41 | 0.26 | 21.64 | 28.36 |
|  | **GR** | 1 | 14.62 | 19.64 | 0.64 | 12.62 | 17.5 |
|  | **TaSOS1** | 1 | 23.84 | 32.15 | 0.52 | 5.37 | 30.55 |
|  |  |  |  |  |  |  |  |
|  |  |  |  |  |  |  |  |
| **GEMMIZA 9** | **GeneID** | **Control** | **S1** | **S2** | **GABA** | **GABA +S1** | **GABA+S2** |
|  | **NHX1** | 1 | 18.9 | 28.62 | 0.64 | 17.59 | 27.9 |
|  | **DHN3** | 1 | 16.4 | 23.45 | 0.52 | 15.37 | 22.54 |
|  | **GR** | 1 | 19.87 | 27.64 | 0.48 | 19.87 | 25.64 |
|  | **TaSOS1** | 1 | 17.6 | 22.9 | 0.67 | 16.99 | 22.43 |
|  |  |  |  |  |  |  |  |
|  |  |  |  |  |  |  |  |
|  |  |  |  |  |  |  |  |
| **GEMMIZA 10** | **GeneID** | **Control** | **S1** | **S2** | **GABA** | **GABA +S1** | **GABA+S2** |
|  | **NHX1** | 1 | 21.64 | 28.61 | 0.67 | 17.25 | 26.34 |
|  | **DHN3** | 1 | 16.95 | 21.6 | 0.55 | 15.94 | 20.96 |
|  | **GR** | 1 | 20.9 | 26.8 | 0.96 | 16.34 | 18.97 |
|  | **TaSOS1** | 1 | 22.09 | 29.4 | 0.78 | 19.11 | 26.97 |
|  |  |  |  |  |  |  |  |
|  |  |  |  |  |  |  |  |
|  |  |  |  |  |  |  |  |
| **GEMMIZA 11** | **GeneID** | **Control** | **S1** | **S2** | **GABA** | **GABA +S1** | **GABA+S2** |
|  | **NHX1** | 1 | 18.92 | 24.68 | 0.64 | 17.9 | 13.36 |
|  | **DHN3** | 1 | 17.64 | 23.97 | 0.25 | 16.52 | 22.87 |
|  | **GR** | 1 | 19.7 | 25.61 | 0.67 | 18.27 | 23.46 |
|  | **TaSOS1** | 1 | 24.98 | 31.54 | 0.95 | 23.61 | 27.94 |
|  |  |  |  |  |  |  |  |
|  |  |  |  |  |  |  |  |
| **GEMMIZA 12** | **GeneID** | **Control** | **S1** | **S2** | **GABA** | **GABA +S1** | **GABA+S2** |
|  | **NHX1** | 1 | 18.64 | 24.67 | 0.64 | 17.2 | 20.64 |
|  | **DHN3** | 1 | 16.5 | 23.9 | 0.58 | 15.49 | 18.27 |
|  | **GR** | 1 | 18.23 | 26.94 | 0.62 | 16.27 | 21.6 |
|  | **TaSOS1** | 1 | 20.47 | 27.82 | 0.58 | 17.92 | 22.45 |
